# Improving productivity of citramalate from CO_2_ by *Synechocystis* sp. PCC 6803 through design of experiment

**DOI:** 10.1186/s13068-024-02589-z

**Published:** 2024-12-05

**Authors:** Matthew Faulkner, Fraser Andrews, Nigel Scrutton

**Affiliations:** Manchester Institute of Biotechnology and Department of Chemistry, The University of Manchester, 131 Princess Street, Manchester, M1 7DN UK

**Keywords:** Cyanobacteria, *Synechocystis*, Citramalate, Methyl methacrylate, Photosynthesis, Carbon capture

## Abstract

**Background:**

Cyanobacteria have long been suggested as an industrial chassis for the conversion of carbon dioxide to products as part of a circular bioeconomy. The slow growth, carbon fixation rates, and limits of carbon partitioning between biomass and product in cyanobacteria must be overcome to fully realise this industrial potential. Typically, flux towards heterologous pathways is limited by the availability of core metabolites. Citramalate is produced in a single enzymatic step through the condensation of the central metabolites pyruvate and acetyl-CoA; improvements in citramalate productivity can, therefore, be used as a measure of overcoming this limitation. Furthermore, citramalate is a useful biomaterial precursor and provides a route to renewable methyl methacrylate and poly(methyl methacrylate), which is often traded as Perspex or Plexiglas.

**Results:**

Here, we describe a phenomenon where the concerted optimisation of process parameters significantly increased citramalate production in *Synechocystis* sp. PCC 6803. Design of experiment principles were used to determine the optima for each parameter and the interplay between multiple parameters. This approach facilitated a ~ 23-fold increase in citramalate titre from initial unoptimised experiments. The process of scale-up from batch cultures to 0.5, 2, and 5 L photobioreactors is described. At the 2-L scale, citramalate titres from carbon dioxide reached 6.35 g/L with space–time yields of 1.59 g/L/day whilst 5-L PBRs yielded 3.96 ± 0.23 g/L with a productivity of 0.99 ± 0.06 g/L/day. We believe the decrease in productivity from 2-L to 5-L scale was likely due to the increased pathlength and shading for light delivery reducing incident light per cell. However, changes in productivity and growth characteristics are not uncommon when scaling up biotechnology processes and have numerous potential causes.

**Conclusions:**

This work demonstrates that the use of a process parameter control regime can ameliorate precursor limitation and enhance citramalate production. Since pyruvate and/or acetyl-CoA give rise to numerous products of biotechnological interest, the workflow presented here could be employed to optimise flux towards other heterologous pathways. Understanding the factors controlling and thus increasing carbon partitioning to product will help progress cyanobacteria as part of a carbon–neutral circular bioeconomy. This is the first study using design of experiment to optimise overall carbon fixation rate and carbon partitioning to product, with the goal of improving the performance of a cyanobacterium as a host for biological carbon capture.

**Supplementary Information:**

The online version contains supplementary material available at 10.1186/s13068-024-02589-z.

## Highlights

- *Synechocystis* sp. PCC 6803 produced 6.35 g/L of citramalate with a space–time yield of 1.59 g/L/day from carbon dioxide_._
- The rate of citramalate production by *Synechocystis.* sp. PCC 6803 was improved from 0.068 to 1.59 g/L/day, a ~ 23-fold increase.
- The switch between growth and production can be controlled using process parameters in place of complex genetic circuits and chemical inducers.
- Overall carbon fixation rates of *Synechocystis* sp. PCC 6803 photobioreactor cultures were increased from 0.12 g/L/day to 0.77 g/L/day.

**Supplementary Information:**

The online version contains supplementary material available at 10.1186/s13068-024-02589-z.

## Introduction

Cyanobacteria are a phylum of photosynthetic organisms that have long been touted as a key asset in establishing a circular bioeconomy [1]. Certain species have been genetically modified to create products of biotechnological relevance (2–3 butanediol [2], butanol [3], ethanol [4], poly(hydroxybutyrate) (PHB) [5] and lactic acid [6], to name but a few) from carbon dioxide (CO_2_). Such organisms could capture CO_2_ at source from industrial offgasses (fermentation [7], power generation [8,9], steel manufacture [10], biogas upgrading [11]) or from the air and ‘recycle’ it into new, useful products that are currently obtained from unsustainable processes. Whilst companies are scaling processes of this type (e.g. Algaecytes 2,000,000 L Omega-3 facility in Dessau, Germany [12]), we are far from realising the full potential of these technologies.

Several issues limit the economic viability and productivity of cyanobacterial biotechnology, thus preventing its widespread use and scale-up [13, 14]. Foremost, the growth and product accumulation rates of phototrophs are slow compared to heterotrophs or traditional chemical processes [15–17]. This leads to lower space–time yields that require larger process volumes and incur higher costs to achieve an equivalent output. To improve space–time yields in cyanobacteria, metabolic engineering strategies have been employed, which include using efficient enzymes, down-regulating competing pathways, and up-regulating favourable pathways [6, 18]. However, productivity can still be restricted due to a limiting precursor supply [19, 20] or the constraints of carbon partitioning (division of resources between biomass and product accumulation) [19, 21]. Finding a mechanism to alleviate precursor limitation would be beneficial for multiple products, as many heterologous pathways draw on common central metabolites such as pyruvate and acetyl-CoA (Table 1). It was recently suggested that environmental factors could be used alongside metabolic engineering to manipulate metabolism and overcome precursor limitation [4]. This idea has been tentatively explored with experiments testing nitrogen and phosphorus limitation [20], sodium stress cycling [22], high-carbon environments [23], and excess light for biochemical production [24]. As these experiments only investigated at most two environmental factors at a time, the potential for applying multiple factors in concert is yet to be explored.

**Table 1:** A comparison of pyruvate and/or acetyl-CoA-derived products from literature with citramalate production in this study Carbon economy refers to the mg of carbon fixed within the product per litre of photobioreactor culture per day, this was calculated by multiplying the yield by the % of the molecular mass of the product comprised by carbon

Product	Titre (mg L^−1^)	Days of production	Yield (mg L^−1^ day^−1^)	CDW (g L^−1^)	Specific growth rate (gCDW L^−1^ day^−1^)	Productivity (mg gCDW^−1^ day^−1^)	Carbon economy (mg C L^−1^ day^−1^)	References
Ethanol	5500	26	212	–	–	–	*111*	[52]
Lactic acid	2170	30	72	–	–	–	*29*	[53]
3-Hydroxy propionate	837	6	140	–	–	58	*55*	[54]
Butanol	37	8	*5*	–	–	8	*24*	[55]
2,3-Butanediol	430	29	60# *15*	–	–	–	*32*	[2]
3-Hydroxy-butyrate	533	21	*25*	–	–	–	*11*	[56]
Ethanol	2960	3	2010* *(986.6)*	1.62	0.54	240* *(117.8)*	1048* (514)	[45]
Propane	24	2	11	2.02	0.11	12	*10*	[38]
Citramalate *N* = 1	6350	4	1589	0.97	0.24	647	653	This study 2-L DOE (Fig. 2)
Citramalate *N* = 2	3960 ± 233	4	990 ± 58	0.99 ± 0.15	0.25 ± 0.04	414 ± 24	406 ± 25	This study 5-L reactors (Fig. 3)
Citramalate *N* = 3	4120 ± 359	4	1030 ± 90	1.15 ± 0.09	0.29 ± 0.02	430 ± 38	422 ± 37	This study 2-L reactors (Fig. 4)

Example calculations are given in the materials and methods. * Denotes a maximum rate of 2010 mg L^−1^ day^−1^ was reported for the first 24 h that decreased over time, over 72 h the rate was 986.6 mg L^−1^ day^−1^. # Denotes total C4 products, this strain was not only producing 2–3 butanediol. Values that were stated in the accompanying citation in black, values that were calculated by us in blue italics. CDW = cell dry weight

**Fig. 1:**
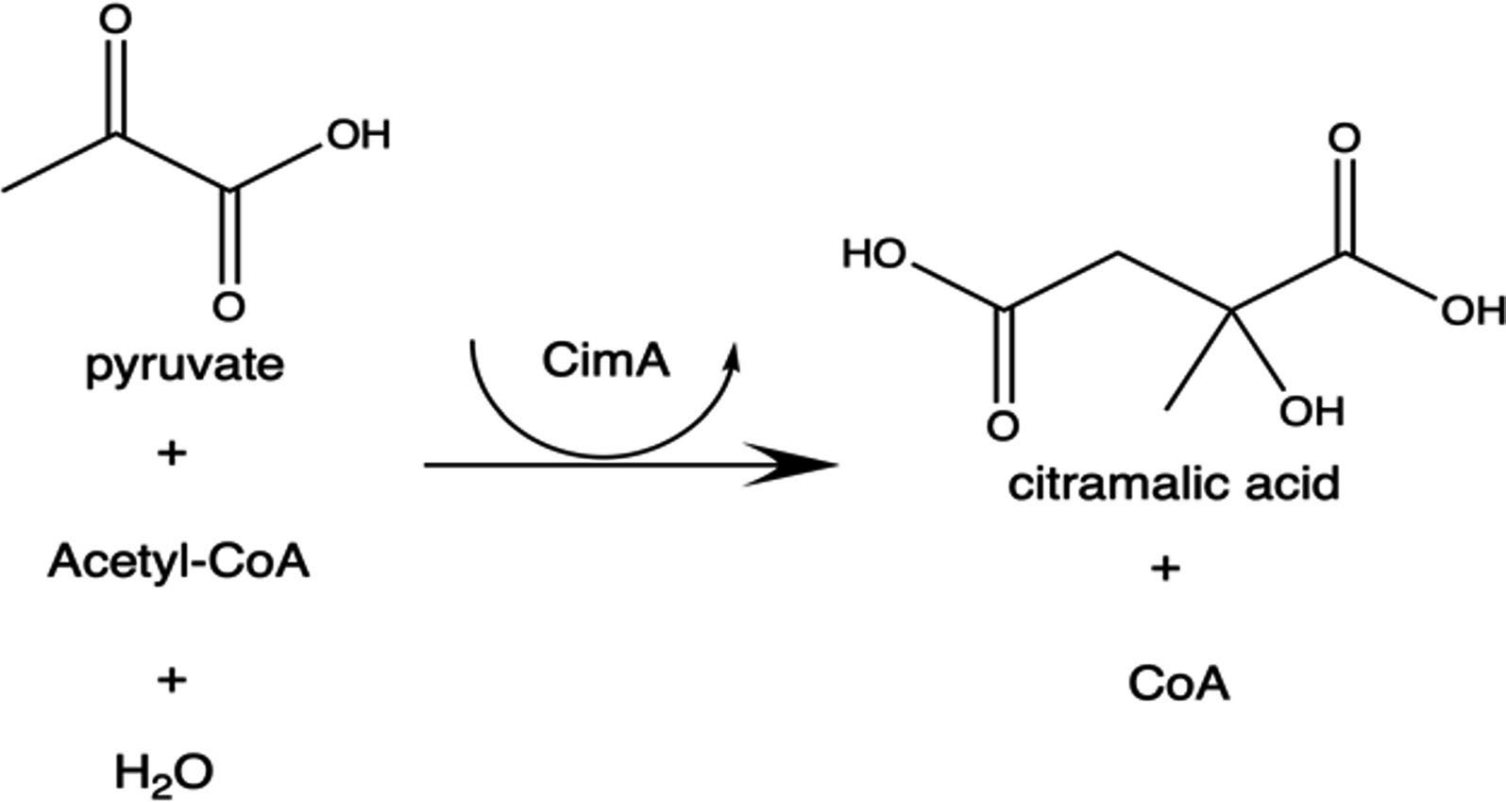
The reaction catalysed by citramalate synthase

To investigate this hypothesis, a strain of *Synechocystis* sp. PCC 6803 was engineered to produce citramalate (citramalic acid, C_5_H_6_O_5_) from pyruvate and acetyl-CoA in a single condensation reaction catalysed by citramalate synthase, E.C. 2.3.3.21, (CimA) [25] (Fig. 1). By applying multivariate process parameters and measuring citramalate titre, it is possible to gauge whether flux through the central metabolites, pyruvate and acetyl-CoA, increases under certain environmental conditions. Furthermore, citramalate is a useful biochemical when converted to methyl methacrylate [26, 27] and polymerised [28] into poly(methyl methacrylate), a high-performance material (often traded as Perspex or Plexiglas).

In this study, nitrogen and phosphorus concentrations in the growth media, CO_2_ concentration mixed with compressed air in the ingas, white light and blue light incident of the surface of the culture are chosen as parameters to be investigated. Each of these induces wholesale changes in the metabolism of *Synechocystis* sp. PCC 6803 [20], and some have been shown to increase bio-product titre [4]. To efficiently navigate this large experimental space and to observe multi-factor interactions, design of experiment (DOE) principles are followed and other important process parameters such as temperature, pH, and the concentration of the other growth medium ingredients were fixed at approximate optima determined in preliminary experiments. This reduces the number of experiments needed, allowing optimisation in large-scale bioreactors with conditions that are directly applicable to a scaled-up process. DOE also enables predictive modelling of productivity under conditions not tested experimentally. If the results of bioreactor experiments show increased citramalate titre under certain environmental conditions, supporting the notion that process conditions can be used to overcome precursor limitation. Moreover, suppose carbon partitioning is affected by the same parameters. In that case, it may be possible to divide batch cultures into growth and production phases, opening the door for parameter-based control of product formation and carbon flux in photobioreactors (PBRs). These findings should have far-ranging applications in cyanobacterial biotechnology, whereby this phenomenon could be used to enhance productivity metrics of bio-chemicals derived from pyruvate and acetyl-CoA (Table 1). This is the first study using DOE to optimise overall carbon fixation rate and carbon partitioning to product, with the goal of improving the performance of a cyanobacterium as a host for biological carbon capture.

## Materials and methods

### Strains used and their construction

*Synechocystis* sp. PCC 6803 [N-1] (GenBank BA000022 *Synechocystis* sp. PCC 6803 DNA, complete genome) obtained from ATCC (ATCC strain 27184) was used as the citramalate production host in this study.

To make the citramalate production plasmid, an engineered Citramalate synthase E.C. 2.3.3.21 mj*cimA*3.7 [29] gene and a mj*cimA*3.7 control inactivated by point mutation [30] (both kind gifts from Dr Joseph Webb in pET20b [31]) were cloned into a modified version of the pAM2991 [32] vector (obtained from Addgene, plasmid 40248), by InFusion [33] (Takara Bio), creating pAM2991*cimA* for production and pAM2991*cimA*_inactive to serve as a negative control. The entire plasmids, insert and backbone, were confirmed by nanopore sequencing (PlasmidSaurus) (Supplementary Table 2). mj*cimA* is thermophilic with an optimum temperature of 70 °C, mj*cimA*3.7 was improved previously by directed evolution through iterative rounds of error-prone PCR increasing *k*_cat_ from mj*cimA*WT ~ 0.36 s^−1^to mj*cimA*3.7 ~ 0.84 s^−1^ at 30 °C [29], mj*cimA*3.7 is henceforth referred to as *cimA* here for simplicity. pAM2991c*imA* and the inactive control were created by linearising pAM2991 at the MCS by restriction digest with EcoRI (New England Biolabs), amplifying *cimA* from pET20b(+)-mjcimA3.7 [31] by PCR and following the standard InFusion protocol detailed in the kit instructions (Takara Bio). The primers used in the construction of and the full plasmid sequence of pAM2991c*imA* are available in the supporting information (Supplementary Tables 1 & 2).

To reduce the conversion of citramalic acid to citraconic acid, 2-methylmalate dehydratase, E.C. 4.2.1.33.4.2.1.35, (*leuC)* was knocked down in *Synechocystis* sp. PCC 6803 by homologous recombination from a modified pAM2991 (Supplementary Fig. 1), creating (*Synechocystis*_D*leuC)* (Supplementary Table 2). *Synechocystis* has one copy of leuC (Genbank ORF_ID:“sll1470”, protein_id = “BAA18738.1”, from base pairs 3,406,416 to 3,407,822 in Genome:BA000022.2) [34]. pAM2991D*leuC* was created by linearising pAM2991 by PCR, amplifying 800-bp flanking regions either side of *leuC* from the *Synechocystis* genome to act as homology arms and a chloramphenicol resistance ‘*cmR’* marker by PCR and following the standard InFusion protocol detailed in the kit instructions to insert all 3 fragments into the linearised pAM2991. The correct construction of the plasmid was confirmed by nanopore sequencing (PlasmidSaurus). The primers used in the construction of the full plasmid sequence of pAM2991D*leuC* are available in the supporting information (Supplementary Tables 1 & 2). pAM2991D*leuC* was transformed into *Synechocystis* sp. PCC 6803 by natural transformation, [35] after overnight growth in liquid culture with chloramphenicol cells were plated onto non-selective BG11 agar plates and then replica plated onto selective plates containing either spectinomycin or chloramphenicol at 20 mg/ml. Only colonies that grew on the non-selective and chloramphenicol-containing plates but not the spectinomycin-containing plate were taken forward and screened by colony PCR (Supplementary Fig. 2). Cells with pAM2991D*leuC* plasmid present were thereby excluded as the plasmid confers spectinomycin and chloramphenicol resistance and the genome-integrated fragment confers only chloramphenicol resistance, and this was also ruled out through the PCR screening. Without any functional LeuC*, Synechocystis* should have no viable pathway for the synthesis of leucine and should therefore be a leucine auxotroph. Leucine auxotrophy was not observed in D*leuC* genotypes, nor were fully segregated knock outs observed, the D*leuC* strain appears to be heterogenous for WT and knock-out despite repeated subculturing on selective media (Supplementary Fig. 2). *Synechocystis_*D*leuC* was not segregated with leucine supplementation thus full segregation would be fatal; *Synechocystis* is polyploid [36] therefore we determined the D*leuC* strain was knocked-down not knocked-out. Genomes were extracted using a kit (Monarch Spin gDNA Extraction Kit) and quality checked by NanoDrop (NanoDrop 2000) and Qubit (Qubit dsDNA BR Broad-Range Assay Kit) then sent for whole genome sequencing by Oxford Nanopore (standard bacterial genome, Plasmidsaurus), genomes were assembled using Flye v2.9.1. These assemblies were used to compare *Synechocystis_*D*leuC* and *Synechocystis_*WT, both consensus genomes were identical indicating the predominant genotype in the *Synechocystis_*D*leuC* heterogeneric population was WT (Supplementary Fig. 3). This D*leuC* knock-down improved citramalate accumulation without impacting significantly upon growth (Supplementary Fig. 5). Optimisation of the ratio of WT to D*leuC* chromosomes and thus the strength of the knock-down was outside the scope of this study.

pAM2991*cimA* and pAM2991*cimA*_inactive were transformed into wild-type *Synechocystis* sp. PCC 6803 and the ∆*leuC* knock-down variant by natural transformation [35], making the final strains *Synechocystis*_∆*leuC* and *Synechocystis*_∆*leuC*_pAM2991*cimA*. Colonies from selective BG11 agar plates, working concentrations of 20 μg/ml spectinomycin and/or chloramphenicol, were screened for the *cimA* insert by colony PCR using bespoke primer pairs to amplify the site of interest premixed in GoTaq green master mix (Promega, UK). Insertion of *chlR* in place of *leuC* in the knock-down strain was confirmed by similar PCR at the insertion site and sanger sequencing of the PCR product. *Synechocystis* sp. PCC 6803 samples were resuspended in 10 μL MQ H_2_O in PCR tubes and incubated at 98 °C for 10 min to release DNA before centrifugation to pellet cell debris, the supernatant was used as template DNA for the PCR. Once verified, 5 independent colonies each with the presence of pAM2991*cimA* confirmed by PCR (Supplementary Fig. 2), these 5 were assayed for citramalate production under standard photosynthetic growth conditions and provide the 5 biological replicates used in this study (Supplementary Fig. 5).

### Detection and quantification of citramalate

Citramalate was detected by high-pressure liquid chromatography (HPLC), and culture samples were filtered through a 0.22 μm syringe filter into HPLC vials (AGILENT 2 ml Vial Screw Cap Clear HPLC 51832068) and stored immediately at − 20 °C until analysis. Samples were thawed on ice and placed in a refrigerated autosampler tray at 4 °C. 10 μL injections were analysed isocratically on an Agilent Hi-Plex H column (300 × 7.7 mm; 2.5 mM H_2_SO_4_) at 60 °C with a flow rate of 0.7 ml min^−1^ for 25 min and detected by refractive index detection (RID) and UV–Vis via diode array detector (DAD). Citramalate was quantified against a standard curve of known concentrations of an authentic analytical quality (> 99%) standard (Sigma, UK). Typical RID and DAD traces and a typical standard curve are shown in the supporting information (Supplementary Fig. 4).

### Growth medium

BG11 [37] growth media as per the University of Texas (UTEX) recipe (https://utex.org/products/bg-11-medium?variant=30991786868826) was used for cultivation and maintenance of all strains. A 100 × stock solution was added to tap water and sterilised by autoclave of all except K_2_HPO_4_ and NaHCO_3_ which were added post autoclave immediately before use via a 0.22-μm syringe filter under sterile conditions. Selective media had working concentrations of 20 μg/ml spectinomycin and/or chloramphenicol added as required immediately before inoculation under sterile conditions via a 0.22-μm syringe filter.

### Batch-scale cultures

Batch cultivations were conducted in Nunc EasY Flasks (Thermo Fisher UK) of appropriate volumes, 1–10 ml culture volumes in 25 ml flasks and 10–25 ml cultures in 50-ml flasks. Flasks were incubated in Infors HT Multitron orbital shaking incubators at 34 °C agitating at 140 RPM (Unimax 1010, Heidolph) with 40 µmol photons m^−2^ s^−1^ warm white light (colour temperature 3000 K) for subculturing and growth of stock cultures. For maintenance and short-term storage, culture flasks were incubated in an Algaetron (Photon System Instruments, Czech Republic) without shaking at 30 °C with 20 µmol photons m^−2^ s^−1^ warm white light (colour temperature 3000 K). For growth on BG11 agar for transformations and picking single colonies, plates were incubated in an Algaetron (Photon System Instruments, Czech Republic) without shaking at 30 °C with 80 µmol photons m^−2^ s^−1^ warm white light (colour temperature 3000 K).

### Flat-panel photobioreactor

For preliminary experiments, 515 ml flat-panel PBR (FMT150, photon system instruments) at the Manchester Institute of Biotechnology, University of Manchester, with 400 ml culture and 115 ml headspace were used, as previously described [38]. pH control was delivered by two peristaltic pumps supplying 1 M NaOH in 2xBG11 and 1 M HCl in 2xBG11 with setpoints of 7.5 and 8.5, respectively. Aeration and mixing were achieved via delivery of 2 VVM compressed filtered air via a sparge at the bottom of the culture in a set-up akin to an airlift reactor. Blue light was delivered by an internal 0–1650 µmol photons m^−2^ s^−1^ 420 nm LED panel, the intensity of the LED array could be set in the reactor control software. Warm white light was delivered by an externally mounted LED 0–220 µmol photons m^−2^ s^−1^ (MWWHL4, Thor Labs, USA), the intensity corresponds to that measured by a light meter transmitted through an empty vessel.

### Parallel stirred tank photobioreactors

The DOE was conducted in 8 × 2 L parallel PBRs (DASGIP, Eppendorf, Germany) at the FlexBio facility in Heriot-Watt University Edinburgh. An 18-data point definitive screening design was made to be run over a series of 3 experiments using 6 vessels, defined in the design as 3 blocks, with 1 backup vessel and 1 control vessel. These first 18 data points were used to inform the variable ranges for a final fourth block of 6 data points to complete a 24-data point design. CO_2_ (the concentration of CO_2_ in the ingas supplemented on top of compressed air), nitrogen (the concentration of NaNO_3_ in the BG11 medium), phosphorous (the concentration of K_2_HPO_4_ in the BG11 medium), white light (the intensity of warm white light incident on the surface of the vessel) and blue light (the intensity of actinic blue light (420 nm) incident on the surface of the vessel) were included as input variables. Optical density at 680 nm (OD 680 nm—the peak absorbance of chlorophyll a), optical density at 720 nm (OD 720 nm—a measure of turbidity and therefore cell biomass), and the concentration of citramalate were measured as continuous outputs. Endpoint cell dry weight (CDW) was also measured. Warm white light and blue light were delivered by LED strips (ZFS-8500HDWW & ZFS-85000HD-B, JKL UK) attached directly to the reactor, the length of the LED strip used determined light intensity. Light intensity per cm of LED strip added was measured by a light meter in the centre of the vessel when empty and used to calculate the correct intensity as per the DOE, thus the incident light intensity values correspond to the midpoint of an empty vessel. CO_2_ concentration in the ingas was maintained by the DASGIP controller gas mixer and CO_2_ probes in the ingas and offgas for continuous measurement. pH control setpoints of < 8.5 and > 7.5 maintained approximately pH 8 with 6 M HCl and 6 M NaOH added via peristaltic pumps. Compressed air, mixed with CO_2_ as required, was supplied at 0.5 VVM, ingas CO_2_ is described in % supplemented into compressed air (% v (CO_2_)/% v (air)); i.e. 4% CO_2_ is constituted of 4% CO_2_ and 96% and air, respectively.

### Design of experiments approach

The statistical software package JMP (JMP^®^, Version *Pro 16*. SAS Institute Inc., Cary, NC, 1989–2023) was used for planning, design, analysis, and modelling of the DOE conditions and resulting data. Parameters tested in the DOE were [CO_2_] in the in gas (% v (CO_2_)/% v (air)), [NaNO_3_] (mM) in the medium, [K_2_HPO_4_] (mM) in the medium, white light intensity (µmol photons m^−2^ s^−1^) and blue light (420 nm, “actinic blue”) intensity (µmol photons m^−2^ s^−1^). Ranges for these parameters were set based on data from FMT150 experiments to determine minimum and maximum values with viable growth (data not shown). The dataset was modelled with Neural, K Nearest Neighbours, Bootstrap Forest, and Support Vector Machines. The Support Vector Machines predictive model fitted our experimental data best with the highest R^2^ of 0.49. The predicted data generated by the Support Vector Machines model was validated by experiments in triplicate in 2-L Fermac vessels (Electrolab, Germany) at the University of Manchester. pH control with setpoints of < 8.5 and > 7.5 maintained approximately pH 8 with 6 M HCl and 6 M NaOH added via peristaltic pumps. Agitation was from a single Rushton-type impellor at the midpoint of the culture maintained at 100 RPM. Aeration was from compressed air, mixed with CO_2_ as required, at 0.5 VVM, ingas CO_2_ is described in % supplemented into compressed air (% v (CO_2_)/ % v (air)).

### Stirred tank photobioreactors

The scaled-up repeats of the highest citramalate titre DOE condition were conducted in a 5-L BioFlo 120 (double glass-walled, water jacketed, direct drive, vessel B120AVB006, Eppendorf, Germany) at the Manchester Institute of Biotechnology, University of Manchester. Warm white light and blue light were delivered by LED strips (ZFS-8500HDWW & ZFS-85000HD-B, JKL UK) attached directly to the reactor outer glass jacket. pH was maintained at 8 with 6 M HCl and 6 M NaOH added via peristaltic pumps with a 0.5 dead band. Mixing was from two Rushton-type impellors at 100 RPM and aeration was from compressed air, mixed with CO_2_ as required, at 0.5 VVM, ingas CO_2_ is described in % supplemented into compressed air (% v (CO_2_)/ % v (air)).

### Carbon fixation and partitioning calculations

Carbon fixation was measured indirectly by measuring CDW and citramalate accumulation. The difference between CO_2_ in the ingas and outgas measurements was not used as a portion of the CO_2_ difference observed is not fixed as either biomass or product and goes to maintenance and other cellular activity. Carbon partition was calculated based on literature values for *Synechocystis* sp. PCC 6803 element analysis, 51.38% carbon [39]. For citramalate, mass = 146.10 g/mol, mass carbon = 60.05, carbon = 41.10%. Therefore, the mass of carbon fixed as biomass was calculated by multiplying endpoint CDW measurements by 0.5138. The mass of carbon fixed as citramalate was calculated by multiplying endpoint PBR values, derived from HPLC, by 0.411.

## Results and discussion

### Construction and validation of the citramalate-producing *Synechocystis* PCC6803 strains

To create a highly productive citramalate chassis, two strains were developed. *Synechocystis*_pAM2991*cimA* contained a plasmid for the over expression of *cimA*, whereas *Synechocystis*_∆*leuC*_pAM2991*cimA* combined this plasmid with the knock-down of *leuC* to reduce the conversion of citramalate to citraconic acid (Supplementary Fig. 1). Batch cultures were grown for 96 h 10 ml culture in 25 ml Nunc EasY flasks at 34 °C agitating at 140 RPM with 40 µmol photons m^−2^ s^−1^ warm white light before citramalate concentration in the culture medium was measured by HPLC. Cultivation in batch cultures showed that *Synechocystis*_∆*leuC*_pAM2991*cimA* accumulated the most citramalate at 238 ± 32 mg/L, significantly more than *Synechocystis*_pAM2991*cimA* (152 ± 37 mg/L), by 2-tailed t-test assuming equal variances (p = 0.037) (Supplementary Fig. 5). Citramalate accumulation was not observed above the detection limit of our HPLC method in the control strains without *cimA* overexpression, or with the inactive *cimA* variant [31] control, inserted in pAM2991 (Supplementary Fig. 5). The comparison of the growth of WT, *cimA* expressing, and inactive *cimA* expressing *Synechocystis* shows there is no significant difference in OD680 nm and/or OD720 nm due to either the expression of *cimA* (*Synechocystis* vs *Synechocystis_*pAM2991*cimA*_inactive) or activity of CimA (*Synechocystis_*pAM2991*cimA*_inactive vs *Synechocystis_*pAM2991*cimA*_active) or both (*Synechocystis* vs *Synechocystis_*pAM2991*cimA*_active) single-factor Anova, *p* = 0.294. Citramalate tolerance was assayed and the half maximal effective concentration (EC50) was ~ 11.87 ± 0.20 g/L showing no significant difference between sub-strains (*Synechocystis_*WT ~ 11.94 ± 1.50, *Synechocystis*_∆*LeuC* ~ 11.65 ± 1.44, *Synechocystis*_pAM2991*cimA* ~ 12.10 ± 1.37, *Synechocystis*_∆*LeuC*_pAM2991*cimA* ~ 11.78 ± 1.25 g/L; single-factor Anova, *p* = 0.344) (Supplementary Fig. 6). Because *Synechocystis*_∆*LeuC*_pAM2991*cimA* had a sufficiently high citramalate tolerance, and reliably accumulated enough citramalate for the purposes of this study, the strain was used for all further experiments.

### Identification of environmental factors for increased citramalate production using design of experiment principles

To investigate whether process parameters could be optimised to increase citramalate yield, a set of 2-L PBR experiments were performed where growth and titre were monitored. To efficiently explore combinations of experimental parameters (nitrogen, phosphorus, CO_2_, white light, and blue light) DOE principles were employed as a custom design based on a definitive screening approach, with midpoints for quadratic effects being used [40]. This allowed multiple variables to be changed in each bioreactor run, details of which can be found in Table 2. Other key process factors, temperature and pH, Iron and thiosulfate concentration in growth medium were fixed at approximate optima determined by preliminary experiments in 0.5-L flat-panel photobioreactors (FMT150, PSI), as were the ranges for the variables tested in the DOE. Light and nutrient variables were gradually increased and decreased until no growth was observed for 24 h, these values signified the bounds of the variables in the DOE. (FMT150 data not shown).

**Table 2:** Summary of the DOE identifiers number, block and vessel (columns 1–3), inputs, ingas CO_2_ in % supplemented into compressed air, i.e. 0% is ambient and 4% is 96% ambient with 4% CO_2_ (4% v (CO_2_)/96% v (air)), blue light incident on the surface of the culture in µmol photons m^−2^ s^−1^, white light incident on the surface of the culture in µmol photons m^−2^ s^−1^, phosphate in concentration added to the starting growth medium mM and nitrate in concentration added to the starting growth medium mM, (columns 4–8) and outputs, endpoint citramalate titre, endpoint OD 680 nm and endpoint OD 720 nm (columns 9–11)

#	Block	Run (R) and vessel (V)	CO_2_ (%)	Blue light (µmol photons m^−2^ s^−1^)	White light (µmol photons m^−2^ s^−1^)	Phosphate (mM)	Nitrate (mM)	Citramalate titre (g/L)	OD 720 nm	OD 680 nm	CDW (g/L)
1	1	R1V2	4	400	400	0.69	45	2.25	6.02	7.02	1.73
2	1	R1V7	0	0	400	0.38	45	2.39	4.15	5.21	1.28
3	1	R1V3	4	0	100	0.07	4.5	1.38	4.05	4.76	1.17
4	1	R1V5	8	400	100	0.38	4.5	1.99	3.47	4.31	1.06
5	1	R2V2	4	200	250	0.38	29	0.55	2.42	3.04	0.75
6	2	R2V3	4	200	250	0.38	29	0.42	4.29	5.36	1.32
7	2	R1V8	0	0	100	0.69	29	2.25	3.71	4.69	1.15
8	2	R1V6	8	400	400	0.07	29	2.15	3.38	4.24	1.04
9	2	R2V5	0	200	400	0.07	4.5	0.27	2.88	3.63	0.89
10	2	R2V6	8	200	100	0.69	45	0.62	2.20	2.70	0.66
11	3	R2V7	8	0	400	0.69	4.5	0.52	2.74	3.46	0.85
12	3	R2V1	0	400	100	0.07	45	0.54	3.07	3.91	0.93
13	3	R3V7	12	600	600	0.07	4.5	4.56	3.02	3.61	0.92
14	3	R3V8	12	0	600	0.07	4.5	4.99	2.98	3.82	0.92
15	3	R3V5	8	400	400	0.07	45	6.35	3.09	3.88	0.97
16	3	R3V6	8	0	250	0.07	45	1.9	2.73	3.28	0.84
17	3	R3V2	0	400	250	0.69	4.5	1.35	3.03	3.74	0.93
18	3	R3V3	4	200	250	0.38	29	0.95	2.82	3.46	0.89
19	4	R4V3	4	600	600	0.07	105.6	1.05	2.89	3.48	0.86
20	4	R4V2	4	100	200	0.15	45	1.28	3.41	4.18	1.03
21	4	R4V6	8	200	400	0.03	29	1.1	2.95	3.94	0.97
22	4	R4V7	6	200	400	0.03	45	0.95	2.69	3.60	0.88
23	4	R4V5	10	400	400	0.07	105.6	1.11	3.06	3.85	1.02
24	4	R4V8	12	600	600	0.07	29	2.84	3.49	4.40	1.08

**Fig. 2:**
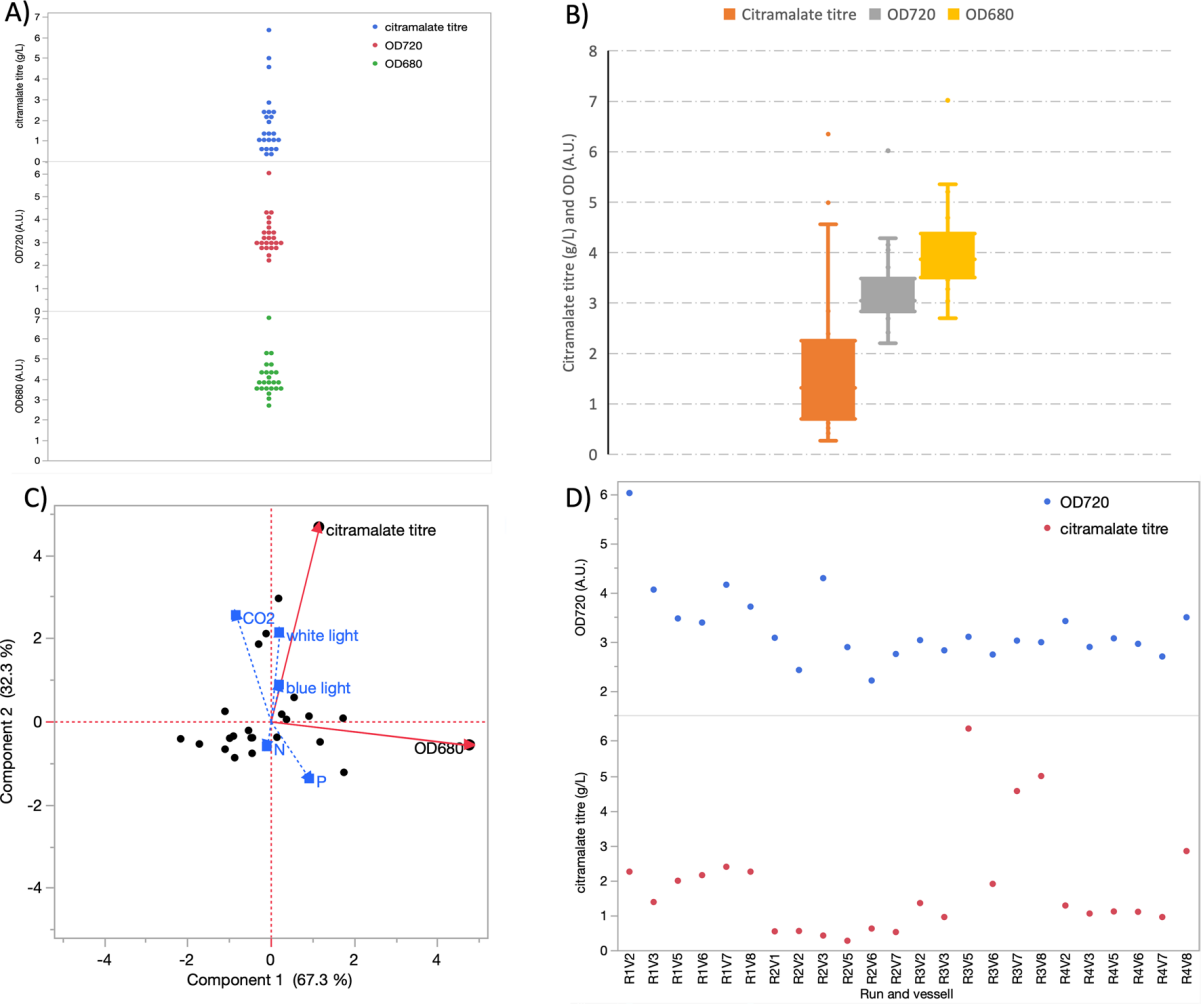
DOE endpoints, **A** a scatter plot and **B** a box plot, showing the final citramalate concentration, OD 720 and OD 680 nm of the 24, 2-L, reactors. **C** Principal component analysis displayed as a biplot. Blue light white light and CO_2_ are the main factors correlating with citramalate titre whilst nitrate and phosphate correlate with growth. **D** A scatter plot of the OD 720 nm and titre of each individual vessel

A wide range of growth, citramalate accumulation, and carbon fixation rates were observed across the DOE, with culture density ranging from 2.20 to 6.02 (OD 720 nm A.U) ~ 2.7-fold, and citramalate from 0.27 to 6.35 g/L, ~ 23-fold between R3V2 and R3V5 (Fig. 2 and Supplementary Fig. 7). Principal component analysis (PCA) revealed that high white light, blue light and CO_2_, improved citramalate titre and productivity, and that conversely low nitrate and phosphate improved citramalate titre and productivity (Fig. 2C). High nitrate & phosphate increased OD 720 and OD 680 nm whilst low appeared to limit growth and decreased OD, suggestion nutrient limitation. These multi-factor correlations are represented in contour plots (Supplementary Fig. 8). A clear optimum was observed around 8% CO_2_ (8% v (CO_2_)/ 92% v (air)), 400 blue and 400 white μmol (photon) m^−2^ s^−1^, 0.076 mM K_2_HPO_4_ and 52.8 mM NaNO_3_. These observations suggest the combination of low nitrate and low phosphate with high light intensity and high carbon availability increases citramalate productivity.

### Reproducing citramalate titres and validating the support vector machine model

**Fig. 3:**
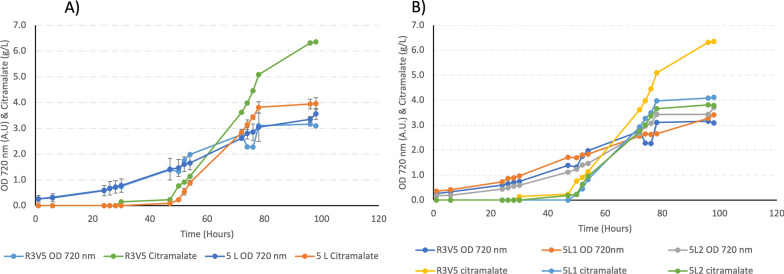
Reproducing the optimum citramalate titre from the DOE. These plots represent duplicates of the experimental maximum citramalate titre from the DOE (R3V5) in 5-L photobioreactors (5L1 and 5L2) both with 8% CO_2_ (8% v (CO_2_)/92% v (air)), 400 mmol (photon) m^−2^ s^−1^ blue and 400 mmol (photon) m^−2^ s^−1^ white light irradiation, 0.076 mM K_2_HPO_4_ and 52.8 mM NaNO_3_. **A** A scatter plot of the of the OD 720 nm (A.U) and titre (g/L) from the most productive experimental DOE condition (R3V5) and repeats treated as a triplicate with the mean plotted and error bars showing 1 SD. **B** The same data split by individual photobioreactor experiment not as a triplicate, until ~ 80 h all 3 growth and production trends were similar, after ~ 80 h the repeats in BioFlo120 produced less citramalate than the DOE vessel. These three photobioreactors were started independently from independent seed cultures with OD 720 nm measurements as follows R3V5, seed = 3.572, starting = 0.261, 5L1, seed = 3.811, starting = 0.356, 5L2, seed = 2.164, starting = 0.174

To test the reproducibility and robustness at scale of the DOE-optimised condition, a set of duplicate experiments, tracking growth and citramalate titre, were performed in a 5-L Eppendorf BioFlo 120 bioreactor (Fig. 3). The 5-L reactors were run under optimum conditions of the DOE, 8% CO_2_ (8% v (CO_2_)/ 92% v (air)), 400 blue and 400 white μmol (photon) m^−2^ s^−1^, 0.076 mM K_2_HPO_4_ and 52.8 mM NaNO_3._ The growth kinetics in the 5-L reactor were similar to the original DOE, achieving a biomass yield when considered as triplicate with a reasonable standard deviation and standard error (OD 720 nm 3.41 ± 0.31 SD, 0.27 SE). Citramalate accumulation was similar for the first 3 days of production but was reduced from ~ 80 h onwards compared to the DOE; if the DOE value and duplicate 5 L values are considered as a triplicate, recorded titres show a high standard deviation and standard error (HPLC 4.75 g/L ± 1.40 SD, 0.80 SE) (Fig. 3). The ~ 80 h decrease at 5-L scale may be due to the larger vessel size which increases light path length through the culture leading to increased shading and reduced photon uptake per cell, particularly at a higher culture density in the later stage of the experiment [41]. This may induce a light-limited state for cells which limits carbon fixation capacity and therefore product titre [42]. Although there are numerous potential reasons for a process operating differently when the scale is increased by 2.5 ×, scale-up challenges of biotechnology processes have been discussed extensively in literature for decades [43], and the exact reason for the decrease in productivity in this case is not clear and will be the subject of future investigation.

**Fig. 4:**
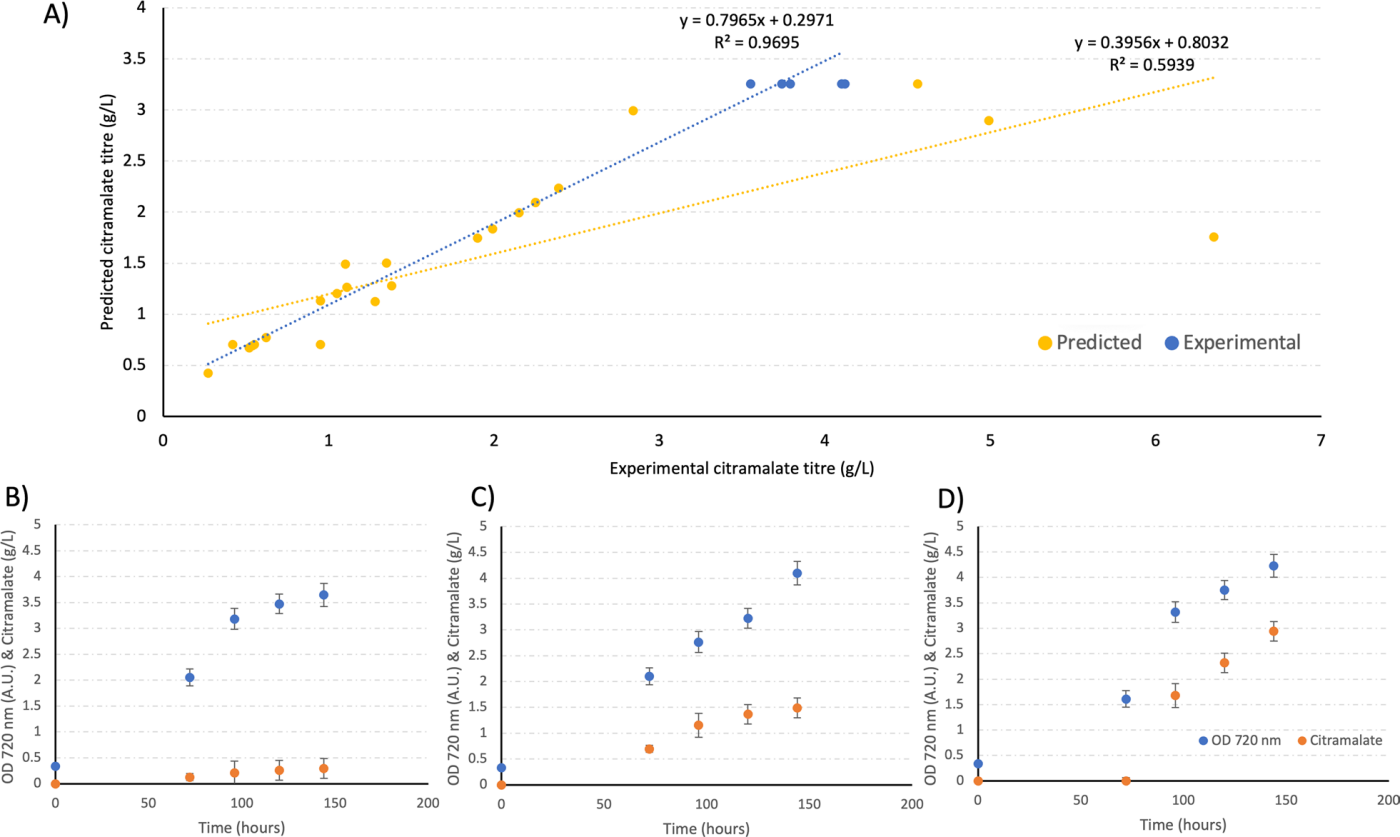
Predicted data from the DOE model vs DOE experimental citramalate titre data, **A** linear fit on all DOE data points (yellow), DOE data (yellow) including repeats of the maximum (blue) in 2 L (*n* = 3) and 5 L (*n* = 2) vessels to experimentally validate the prediction. A linear relationship between predicted and experimental citramalate titre indicates the model is in agreement with the experimental findings. There is a clear linear relationship between predicted and experimental citramalate titre between 0 and 3 g/L, there is a plateau at ~ 3–5 g/L therefore, a less clear linear relationship, and a decrease after ~ 5 g/L giving a non-linear relationship, suggesting the predicted citramalate titre and experimental titre are similar, in agreement, and thus the model is accurate at predicting titres up to ~ 3 g/L. The second linear fit (blue) on the repeat and DOE data points excluding the highest 2 points from the DOE with a much-improved fit vs panel **A**. **B** A repeat of the minimum citramalate titre prediction (similar to DOE vessel R2V3) citramalate titre (orange) and OD 720 nm (blue) displayed in a scatter plot with error bars representing 1 SD. **C** A repeat of the median citramalate titre prediction (similar to DOE vessel R4V7), and **D**) a repeat of the maximum citramalate titre prediction (similar to DOE vessel R3V5) both in a similar scatter plot

A predictive support vector machines model [44] was constructed with the 24 data points representing each of the 2-L reactors of the DOE (Fig. 4). The weighting of model produced in JMP with our data is available in the supplementary information (Supplementary Fig. 9). To experimentally validate the model, the conditions predicted to yield the maximum, median, and minimum titres were tested in 2-L Electrolab Fermac vessels (Fig. 4). The minimum condition was 3% CO_2_ ingas (3% v (CO_2_)/ 97% v (air)), 250 blue and 200 white μmol (photon) m^−2^ s^−1^ light, 17.6 mM NaNO_3_ and 0.23 mM K_2_HPO_4_ BG11, the median condition was 8% CO_2_ (8% v (CO_2_)/92% v (air)), 200 blue and 400 white μmol (photon) m^−2^ s^−1^, 0.16 mM K_2_HPO_4,_ 52.8 mM NaNO_3_, and the maximum condition was 8% CO_2_ (8% v (CO_2_)/92% v (air)), 400 blue and 400 white μmol (photon) m^−2^ s^−1^, 0.076 mM K_2_HPO_4_ and 52.8 mM NaNO_3_. These data corroborate the model and fit the same linear trend line of best fit within 95% confidence (R^2^ = 0.96) for predicted vs actual citramalate titre up to ~ 4 g/L (Fig. 4B), including the highest 2 data points of the DOE decreases the quality of the fit (R^2^ = 0.59, Fig. 4A). This suggests factors or complex interactions not accounted for in the model might explain the highest titres, such as interactors of 3 or more parameters and factors other than CO_2_, light, K_2_HPO_4_ and NaNO_3_. Up to 4 g/L the model was able to accurately predict the citramalate titre based only on the 5 DOE parameters, representative minimum, median, and maximum predictions were tested and gave the expected titres (minimum predicted 0.2 g/L: actual 0.293 ± 0.045 g/L, median predicted 1.5 g/L: actual 1.49 ± 0.19 g/L, maximum predicted 3 g/L: actual 2.94 ± 0.66 g/L. Figure 4C–E). These corroborating data suggest the phenomenon observed in the DOE where combinatorial parameter optimisation improves citramalate productivity is reproducible. The difference between the maximum observed titre in the DOE (6.3 g/L) and the attempts to reproduce it (3.9 and 4.1 g/L) (Table 1) may be due to differences between the sites where these data were gathered, Heriot-Watt University and The University of Manchester, or bioreactors, Eppendorf DASGIP or Eppendorf BioFlo120, respectively. The data described above, comparing predicted and actual data (Fig. 4), collected at The University of Manchester in the same vessel shows less variation than data collected across two sites and different vessels (Table 1). The interaction of up to two factors was accounted for by quadratic points in our DOE, but the interaction of three or more factors is not. Another factor, such as the incident light profile on any given cell could explain the discrepancy between experimental and predicted data above 4 g/L. Incident light was measured and controlled at the inside surface of the vessel wall, the point at which it first irradiates the culture. A cell moving in the culture medium will transition from maximum irradiance at this point to dark at the centre of the vessel farthest from the vessel wall where it is shaded by the other cells between it and the light source. This will in effect give rise to a pulsed light profile as the cell moves from light to dark and back again as a function of the culture density, the denser the culture the more cells shading thus the lower the irradiance at the dark point, such complex factors were not accounted for in the DOE.

### Environmental factors influence carbon partitioning between product and biomass

**Fig. 5:**
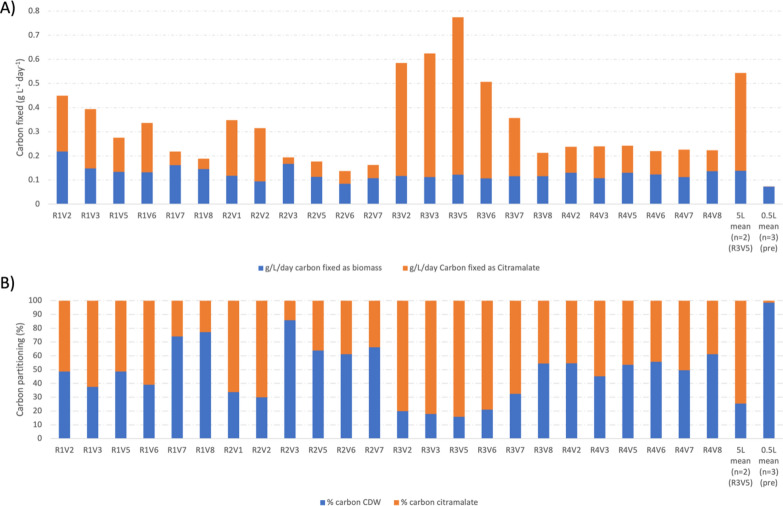
Carbon partitioning across the 24 vessels of the DOE. **A** Rate of carbon fixed as biomass (blue) and citramalate (orange) per day and **B** relative carbon partitioning. R1-4 corresponds to the run and V1-8 corresponds to the vessel, on the far right 5 L corresponds to repeats of the DOE maxima in BioFlo120 (*n* = 2) and 0.5L corresponds to initial experiments in the FMT150 prior to the DOE (*n* = 5). Citramalate represents the major constituent peak in the HPLC data, it is possible other metabolites are released from the cell into the culture medium that were not detected by our HPLC method and were not accounted for in the carbon partitioning calculation

Environmental factors can cause large-scale reorganisation of cyanobacterial metabolism, potentially resulting in differential carbon partitioning values which could be useful for increasing product titres [5]. To investigate this, the carbon content of biomass and product was compared showing that the ratio was affected by the conditions tested in the DOE. Before the DOE, batch cultures and 0.5-L PBRs gave carbon partition values of 98.5 ± 0.63% biomass: 1.5 ± 0.63% citramalate. The range across the DOE conditions was 85.8% biomass: 14.2% citramalate to 15.7% biomass: 84.3% citramalate (Fig. 5). This shift in the high product partition conditions was reproducible in 5-L PBRs 25.4 ± 2.66% biomass: 74.6 ± 2.66% citramalate (*n* = 2). This was accompanied by a significant increase in the carbon fixation rate 0.073 ± 0.0046 g carbon/L/day before the DOE in 0.5-L PBRs, ranged across the DOE 0.137 to 0.774 g carbon/L/day a ~ fivefold change, reproduced in 5-L PBRs 0.544 ± 0.0145 g (carbon)/L/day. A 2 tailed t-test assuming equal variances in the maximum carbon fixation rate observed before and after the DOE (*p* = 8 × 10^–6^) indicates a statistically significant change. Carbon fixation rate was measured indirectly by calculating the carbon content of the biomass and citramalate produced, similar to previous studies [45]; see the Materials and methods.

These results suggest that combinatorial variation of parameters might be a useful tool for process control between growth and production and to upregulate flux through pyruvate, perhaps by overflow metabolism [46] or as part of the stress response [4]. Previous studies have shown the effects of single stresses, sodium stress has up to a 50% increase of soluble carbohydrate [22] and nitrogen limitation leads to a twofold increase in PHB [47]. Improvements from other single stress responses are reviewed by Andrews et al. [4] but no single stress response gives rise to a ~ 23-fold improvement as reported in this study by comparison between the lowest and highest titre in the DOE. It must be noted that the shortcomings of DOE based optimisation include that the results are directly and specifically influenced by the methodology. Therefore, this study revealed the optimal conditions for citramalate production in 2-L Eppendorf DASGIP vessels. When scaling up to 5-L Eppendorf BioFlo120 vessels, consideration needs to be paid to the extrapolation of optimal parameters, and the changes in cellular physiology. Many differences including light pathlength, gas transfer to media, mixing rates, may influence growth rates, nutrient consumption rates, cellular metabolism and citramalate productivity. Extrapolating the optimal parameters directly to a larger bioreactor will likely result in poorer performance, however, this study argues that the changes in cellular metabolism found in both fermenters will still be applicable when scaled up. This necessitates subsequent rounds of DOE optimisation to find the same citramalate productivity at larger scales. The scale-up therefore teaches us about the importance of how parameters and DOE optimisation interact with different photobioreactors.

We suggest that the DOE workflow described here where a common inoculum is split into vessels, grown identically and later switched to the varied production conditions of the DOE, could be used to find optimum conditions for alleviating precursor limitation and optimising a 2-step bioprocess. Here we show that it is possible to modulate carbon partitioning using environmental parameters, allowing *Synechocystis* PBRs to be split into growth and production phases. Previously this control has only been achieved using the complex and potentially genetically unstable molecular biology technique CRISPR interference [48], use of day–night cycles with growth in the light regime and production in the dark regime [49], or complex metabolic engineering such as overexpression of sigma factors in concert with nitrogen limitation [50], or cyclical variation of salt stress [22]. Using process parameters to control carbon partitioning is simpler and offers greater traction for industrial-scale production and could be used in concert with CRISPR interference [48], circadian rhythm [49], and metabolic engineering [50].

Further study is required in industrial-scale PBRs and with industrially applicable strains is required to determine if this could be applied to current cyanobacterial and microalgal processes. Such processes are typically in a different type of reactor with a greater surface area-to-volume ratio to allow for more efficient and cost-effective light delivery at scale, in which this design of experiment workflow and apparent optimum is yet to be tested, e.g. serpentine reactors [51] (Phyco-Flow, Varicon Aqua, Worcester UK). Such reactors have very different fluid dynamics/mixing, how such differences influence this workflow and optimum condition remains to be seen. A DOE approach similar to the one used here would be an efficient way to conduct such a future study.

## Conclusions

The conditions suitable for good growth (specific growth rate > 0.3 g CDW L^−1^ day^−1^), and the conditions for good productivity (> 0.4 g/g CDW /day) (Tables 1 and 2) of *Synechocystis*_∆*LeuC*_pAM2991*cimA* are distinct, thereby separating the growth and production of citramalate into two distinct regimes using light, carbon, and nutrient availability. This enables process control without the need for expensive chemical inducers or more complex two-stage processes in separate vessels. The citramalate production of *Synechocystis*_∆*LeuC*_pAM2991*cimA* was improved using blue light and nutrient limitation. A reproducible optimum was determined around 8% CO_2_ (8% v (CO_2_)/92% v (air)), 800 μmol (photon) m^−2^ s^−1^ illumination with a 50:50 balance of actinic blue:white light, 0.076 mM K_2_HPO_4_ and 52.8 mM NaNO_3_. Proof of concept for cyanobacterial citramalate with high carbon to product fixation rates and improved space–time yields has been shown. A shift in carbon partitioning which could lead to improved space–time yields for other products in future studies has been elucidated.

The coordinated combinatorial application of these parameters has synergistic benefits greater than the application of a single process parameter in our predictive model. A linear relationship fits the predicted vs actual plot up to ~ 4 g/L, suggesting factors, or complex interactions of more than two parameters, not accounted for in the model might explain the highest titres. We plan to investigate this further with proteomics and include relative expression levels of key genes and pathways as parameters in the predictive model and determine if the underlying mechanism is one of stress response and/or overflow metabolism.

Additional parameters optimised in a combinatorial way could further enhance this apparent optimum and yield greater than ~ 23-fold improvements. For example, this could be due to the alleviation of the bottleneck in flux through pyruvate and/or acetyl-CoA as described in numerous previous studies [4]. This improvement may also be related to overflow metabolism and citramalate acting as a carbon sink. The suspected mechanism behind this increase in citramalate productivity of upregulated flux through pyruvate and acetyl-CoA is of interest for further study in *Synechocystis* sp. PCC 6803 and perhaps other more industrially applicable photosynthetic hosts as well.

## Supplementary Information

Additional file 1: Supplementary Fig. 1: Plasmid maps for the two versions of pAM2991 used in this study created in SnapGene. Supplementary Fig. 2: DNA electrophoresis agarose gel images. Lanes that were loaded are numbered in red along the top, empty lanes are unnumbered. A) The sizes and masses of the characteristic bands of Quick-Load 1 kb DNA Ladder, 10 μL of which was loaded in lane 1 in all gel images. B) An agarose gel with samples from colony PCR to confirm the presence of *cimA*. Boil preps of whole *Synechocystis* cells were used as template with the cimA_screen_F & cimA_screen_R primer pair giving an expected size of 1387 bp. Lanes 2–9 of the top gel have independent colonies from BG11 spectinomycin plates used as template, the bottom gel lanes 2–3 has a positive control of pure pAM2991 prior to transformation used as template, lanes 4–5 has a negative control of untransformed *Synechocystis* stock used for the natural transformation*.* C) An agarose gel with samples from colony PCR to confirm the presence of the chloramphenicol resistance marker, all amplified with the leuC_screen_F & leuC_screen_R primer pair expected size of 2896 bp. Lane 2 holds a positive control of the pure pAM2991_Δ*leuC* plasmid, lanes 3–5 hold colony PCRs from independent colonies of *Synechocystis* transformed with pAM2991_Δ*leuC* on a BG11 chloramphenicol plate, lane 6 holds a negative control lanes of untransformed *Synechocystis,* lanes 7–9 hold colony PCR samples from the same 3 independent colonies after replica plating onto a BG11 chloramphenicol and spectinomycin plate. D) An agarose gel confirming the insertion of the chloramphenicol resistance marker into the *Synechocystis* genome. Isolated gDNA was used as template for amplification with the leuC_genome_F & leuC_genome_R primer pair expected size for WT is 5328 bp and Δ*leuC* is 4581 bp. E) An agarose gel showing colony PCRs on whole cell boil prep samples, lane 2 holds a negative control of untransformed *Synechocystis* with both primer pairs*,* lanes 3–6 use the same colonies from lanes 6–7 in, lanes 3–4 used the primer pair leuC_screen_F & cmR_internal_R expected size 1130 bp, lanes 5–6 used the primer pair leuC_screen_R & cmR_internal_F expected size 1230 bp. Supplementary Fig. 3: Consensus genome sequences from Oxford Nanopore dataof *Synechocystis_WT*prior to any of the changes made and *Synechocystis_*Δ*leuC_pAM2991_cimA* as used for production of citramalate from this study mapped to NCBI GCF_000009725.1 as a reference genome, created in SnapGene. Both consensus genomes contain *leuC* 100% identity match to the reference when aligned using Blastn indicating the predominant population is WT. Neither genome contains any significant homology to pAM2991*cimA* when attempting to align with Blastn, indicating the *cimA* expression plasmid has not integrated into the genome of the production strain. There is a slight decrease in coverage and slight indication of contamination in the *Synechocystis_*Δ*leuC_pAM2991_cimA* perhaps indicating the mixed population between Δ*leuC* and WT facilitating the knockdown of *leuC*. The raw sequence read data are available in fastq format as additional files 2 & 3 on OSF https://osf.io/ay52x/ and on Figshare https://doi.org/10.6084/m9.figshare.27170022. Supplementary Fig. 4: Typical UV–vis Diode Array Detectorand Refractive Index Detectorplots and a standard curve for the quantification of citramalate by HPLC. A) An example standard curve 0.1–10 g/L from one citramalate quantification by HPLC, a similar standard curve was generated alongside each batch of samples quantified. B) A typical RID trace from an experimental sample of *Synechocystis* in BG11, the peak at 10.699 min is citramalate, the peak at 7.322 is also present in BG11 only control samples. C) A typical DAD trace from an experimental sample of *Synechocystis* in BG11. Citramalate has no UV absorption so gives signal, there are no peaks other than the one at 6.975 min which is also present in BG11 only control samples. D) A typical RID trace from a standard curve sample of 10 g/L in BG11, the peak at 10.487 min is citramalate. E) A typical DAD trace from a standard curve sample of 10 g/L in BG11. Citramalate has no UV absorption so gives signal, there are no peaks other than the one at 6.823 min which is also present in BG11 only control samples. Supplementary Fig. 5: A box plot of the OD 680 nm & 720 nm, growth, and citramalate titre of the strains tested in this study. Endpoint OD and HPLC measurements after 96 h 10 ml culture in 25 ml Nunc EasY flasks at 34 °C agitating at 140 RPM with 40 µmol photons m^−2^ s^−1^ warm white light, in BG11 medium. 5 independent colonies of wild typeand Δ*leuC*, with and without pAM2991*CimA*, were assayed for citramalate production under standard photosynthetic growth conditions. We observed citramalate production by all of sub-strains with pAM2991*CimA*, *Synechocystis*_Δ*leuC*_pAM2991*CimA* accumulated the most citramalate, significantly more than *Synechocystis*_WT_ pAM2991*CimA* by two-tailed T-test assuming equal variance. We did not observe citramalate accumulation above the detection limit of our HPLC method in controls, strains with no *CimA* or strains with an inactive *CimA* variant [1] insert in pAM2991. Without any functional LeuC*, Synechocystis* should be a leucine auxotroph. We did not observe leucine auxotrophy in Δ*leuC* genotypes, nor did we observe fully segregated knock outs, our strain appears to be heterogenous for WT and knock out despite repeated subculturing on selective media. *Synechocystis_* Δ*leuC* was not segregated with leucine supplementation thus full segregation would be fatal; *Synechocystis* is polyploid [2] thus the Δ*leuC* was knocked-down not knocked-out. This Δ*leuC* knock-down improved citramalate accumulation without impacting upon growth. Optimisation of the ratio of WT to Δ*leuC* and thus the strength of the knock down was outside the scope of this study. Supplementary Fig. 6: Endpoint growth measurementsafter 96 h with citramalate supplemented, left final OD 720 nmand right responseto citramalate supplementation EC50 or 50% decrease in growth is indicated by the red line. The tolerance of each of the sub-strains to exogenous citramalate was determined by measuring growth over 96 h in BG11 supplemented with citramalate 0 – 25 g/L. EC50 was ~ 11.93 g/L. There was no discernible difference in the growth of any sub-strain with citramalate supplements 0–5 g/L. Supplementation of citramalate > 10 g/L began to markedly reduce growth of all sub-strains, and no growth was observed in any sub-strain with > 20 g/L, with no significant difference between OD 720 nm at inoculation and after 96 h for any strainby two-tailed T-test assuming equal variance. Supplementary Fig. 7: The growth in blue OD 720 nm, and citramalate production in orange, curves for all 24 vessels in the DOE. Each plot is labelled top left with the run and vessel ID, for example run 1 vessel 1 is R1V1. The exact condition for each vessel can be found by cross-referencing with Table 1. In the literature where productivity was assessed over time it appears to start high and decrease over time. For example, Namakoshi et al. [3] reported 2010 mg L^−1^ day^−1^ was reported for the first 24 h that decreased over time, over 72 h the rate was 986.6 mg L^−1^ day^−1^. We have observed a similar trend with an initial maximal rate soon after changing conditions evident in most vessels of the DOE, that also tends to decrease over time. Highly productive cultures were also beginning to lose cell viability. Aliquots taken at 96 h from R3V2, R3V3, R3V5, and R3V6 showed little to no growth when spotted onto BG11 agar plates. Similar tests from all other vessels gave clear growth without signs of contamination. Supplementary Fig. 8: Contour plots showing citramalate titre for each possible iteration of 2-factor interaction between CO_2_, blue light, white light, phosphate, nitrate, and OD 720 nm. There are clear minima and maxima and clear interactions between some factors. There is a particularly strong correlation between growth and citramalate titre as indicated by the tight maxima around OD 720 nm ~ 3 in all contour plots. There are clear optima maxima for example when blue and/or white light is high around high CO_2_ ~ 8%, low phosphate ~ 0.2 or 1/5th the concentration of BG 11 medium, low nitrate ~ 0.5–1 or 8.8–17.6 mM. Some plots have no clear maxima show no correlation between the two variables and titre, such as the plot of phosphate, nitrate, and citramalate titre. From these contour plots we can conclude that there are complex multi-factor interactions determining citramalate titre and no single variable tested is determinant for the observed titres. Supplementary Fig. 9: A summary of the models tested in JMP and the weighting of the DOE factors in the equation of the support vector machines model given as ‘T#6’. We made a predictive model to better examine the complex nature of the multi-factor interactions determining citramalate titre, and to extrapolate from the conditions tested experimentally. The experimental data were fitted to all the models available in JMPusing default parameters, excluding any that fit only when excluding 1 or more of our 24 data points as outliers. The support vector machines had the highest R^2^, 0.4884, and the best fit for a predicted vs actual citramalate titre plot. Supplementary Table 1: Primers used in this study. Supplementary Table 2: Plasmid sequences, these sequences were confirmed on plasmids isolated from *Synechocystis* using the “standard high concentration plasmid” sequencing service performed by Plasmidsaurus using Oxford Nanopore Technology with custom analysis and annotation.

## Data Availability

The supplementary information accompanying this article is available in additional file 1 and the genome sequences of the strains used in this study are available in additional files 2 and 3 as a published dataset on OSF https://osf.io/ay52x/ and Figshare 10.6084/m9.figshare.27170022.”
