# Supplementary material for: Improving productivity of citramalate from CO2 by Synechocystis sp. PCC 6803 through design of experiment
Source: Biotechnol Biofuels Bioprod. 2024 Dec 5;17:143. doi: 10.1186/s13068-024-02589-z (PMC11622482; doi:10.1186/s13068-024-02589-z)
Supplement: Supplementary file 1 — Additional file 1: Supplementary Fig. 1: Plasmid maps for the two versions of pAM2991 used in this study created in SnapGene. Supplementary Fig. 2: DNA electrophoresis agarose gel images. Lanes that were loaded are numbered in red along the top, empty lanes are unnumbered. A) The sizes and masses of the characteristic bands of Quick-Load 1 kb DNA Ladder, 10 μL of which was loaded in lane 1 in all gel images. B) An agarose gel with samples from colony PCR to confirm the presence of cimA. Boil preps of whole Synechocystis cells were used as template with the cimA_screen_F & cimA_screen_R primer pair giving an expected size of 1387 bp. Lanes 2–9 of the top gel have independent colonies from BG11 spectinomycin plates used as template, the bottom gel lanes 2–3 has a positive control of pure pAM2991 prior to transformation used as template, lanes 4–5 has a negative control of untransformed Synechocystis stock used for the natural transformation. C) An agarose gel with samples from colony PCR to confirm the presence of the chloramphenicol resistance marker, all amplified with the leuC_screen_F & leuC_screen_R primer pair expected size of 2896 bp. Lane 2 holds a positive control of the pure pAM2991_ΔleuC plasmid, lanes 3–5 hold colony PCRs from independent colonies of Synechocystis transformed with pAM2991_ΔleuC on a BG11 chloramphenicol plate, lane 6 holds a negative control lanes of untransformed Synechocystis, lanes 7–9 hold colony PCR samples from the same 3 independent colonies after replica plating onto a BG11 chloramphenicol and spectinomycin plate. D) An agarose gel confirming the insertion of the chloramphenicol resistance marker into the Synechocystis genome. Isolated gDNA was used as template for amplification with the leuC_genome_F & leuC_genome_R primer pair expected size for WT is 5328 bp and ΔleuC is 4581 bp. E) An agarose gel showing colony PCRs on whole cell boil prep samples, lane 2 holds a negative control of untransformed Synechocystis with both primer p [file 13068_2024_2589_MOESM1_ESM.docx]

**Additional file 1**

**Improving production of citramalate from CO_2_ by *Synechocystis* sp. PCC 6803 through design of experiment**

Matthew Faulkner^*1^, Fraser Andrews^1^, & Nigel S. Scrutton^1*^,

^1^Manchester Institute of Biotechnology, The University of Manchester, 131 Princess Street, Manchester M1 7DN, United Kingdom

^*^Corresponding authors

**Table of contents**

|  |  |  |
| --- | --- | --- |
| **Figures** |  |  |
| Supplementary Figure 1 | Maps of the plasmids used in this study | 2 |
| Supplementary Figure 2 | DNA gel images detailing plasmid construction | 2 |
| Supplementary Figure 3 | Genome sequences of *Synechocystis* and *Synechocystis_*Δ*leuC* | 3 |
| Supplementary Figure 4 | Typical DAD and RID plots and a standard curve for the quantification of citramalate by HPLC | 4 |
| Supplementary Figure 5 | An overview of the strains used in this study | 5 |
| Supplementary Figure 6 | Citramalate tolerance | 6 |
| Supplementary Figure 7 | Growth and citramalate production of the individual DOE vessels | 6-8 |
| Supplementary Figure 8 | Contour plots of two factor interactions | 9 |
| Supplementary Figure 9 | Details of the predictive model | 10 |
|  |  |  |
| **Tables** |  |  |
| Supplementary table 1  Supplementary table 2 | Primers used in this study  Plasmid sequences | 11  12-15 |

**References**

1. Joseph P. Webb^​^, S. Alison Arnold, Scott Baxter, Stephen J. Hall, Graham Eastham and Gill Stephens*.* Efficient bio-production of citramalate using an engineered Escherichia coli strain. *Microbiology* 164, (2017).

2. Zerulla, K., Ludt, K. & Soppa, J. The ploidy level of Synechocystis sp. PCC 6803 is highly variable and is influenced by growth phase and by chemical and physical external parameters. *Microbiology* 162, 730–739 (2016).

3. Namakoshi, K., Nakajima, T., Yoshikawa, K., Toya, Y. & Shimizu, H. Combinatorial deletions of glgC and phaCE enhance ethanol production in Synechocystis sp. PCC 6803. *J. Biotechnol.* 239, 13–19 (2016).

**Additional file: 1 Figures**

**
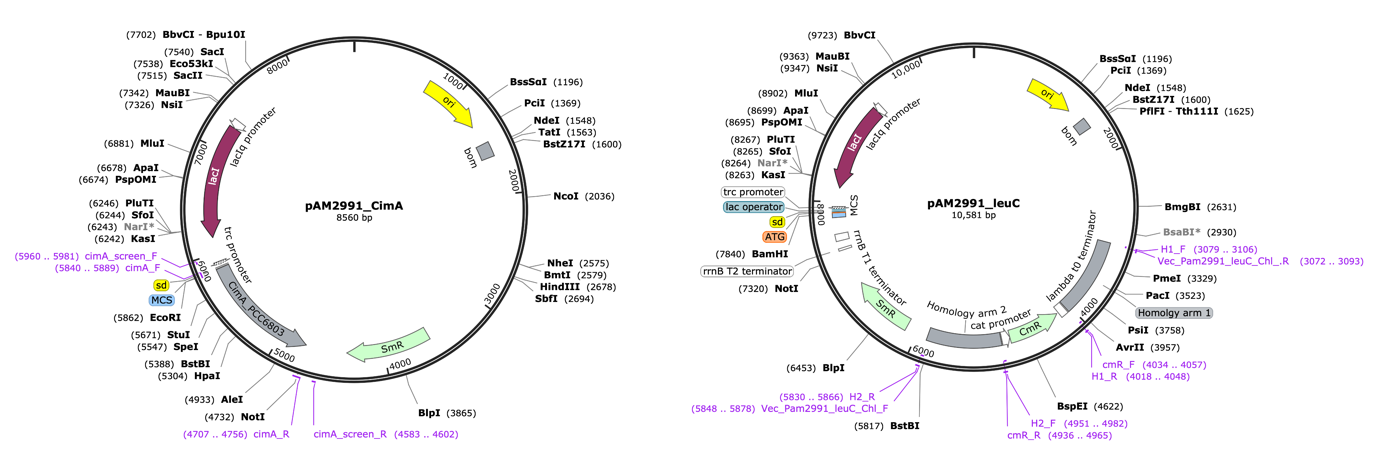
**

**Supplementary Figure 1:** Plasmid maps for the two versions of pAM2991 used in this study created in SnapGene (SnapGene software, www.snapgene.com).

**
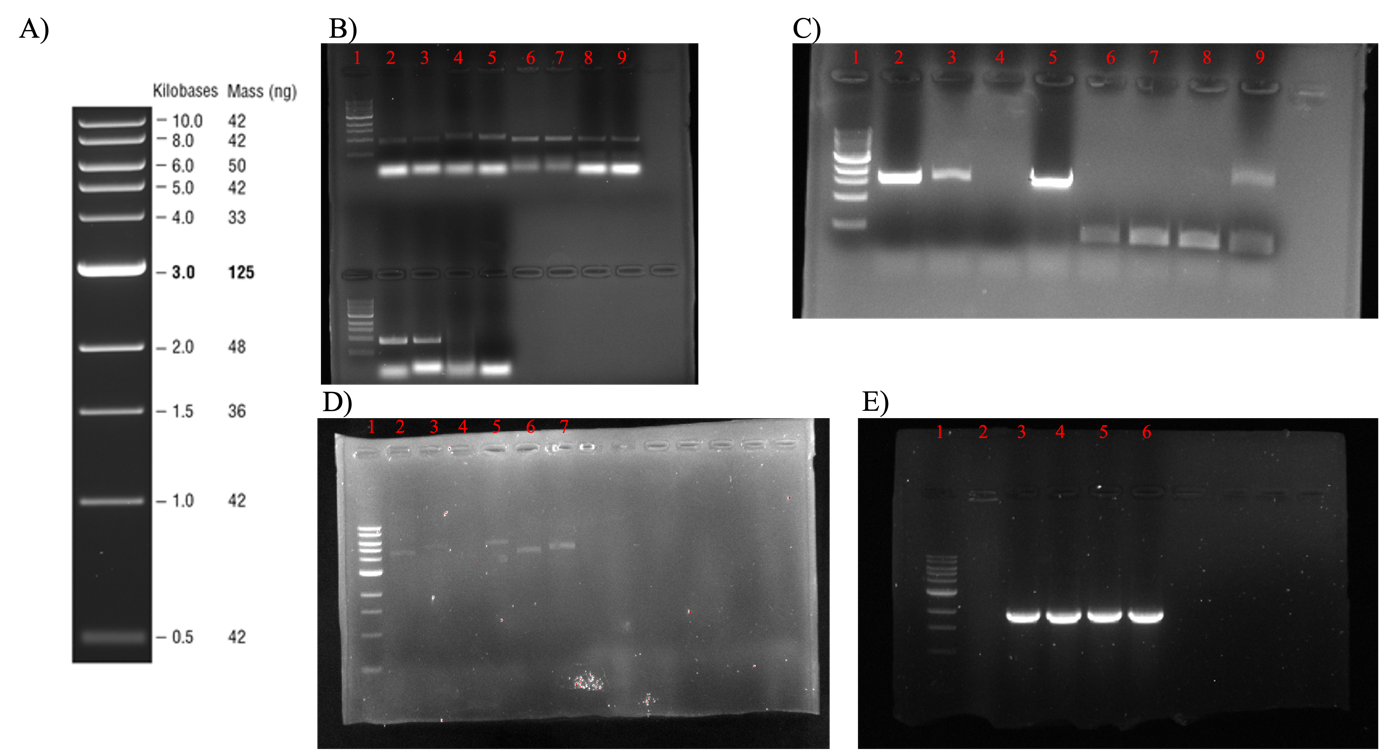
**

**Supplementary Figure 2:** DNA electrophoresis agarose gel images. Lanes that were loaded are numbered in red along the top, empty lanes are unnumbered. A) The sizes and masses of the characteristic bands of Quick-Load 1 kb DNA Ladder (New England Biolabs), 10 μL of which was loaded in lane 1 in all gel images (B-E). B) An agarose gel with samples from colony PCR to confirm the presence of *cimA*. Boil preps of whole *Synechocystis* cells were used as template with the cimA_screen_F & cimA_screen_R primer pair giving an expected size of 1387 bp. Lanes 2-9 of the top gel have independent colonies from BG11 spectinomycin plates used as template, the bottom gel lanes 2-3 has a positive control of pure pAM2991 prior to transformation used as template, lanes 4-5 has a negative control of untransformed *Synechocystis* stock used for the natural transformation*.* C) An agarose gel with samples from colony PCR to confirm the presence of the chloramphenicol resistance marker, all amplified with the leuC_screen_F & leuC_screen_R primer pair expected size of 2896 bp. Lane 2 holds a positive control of the pure pAM2991_Δ*leuC* plasmid, lanes 3-5 hold colony PCRs from independent colonies of *Synechocystis* transformed with pAM2991_Δ*leuC* on a BG11 chloramphenicol plate, lane 6 holds a negative control lanes of untransformed *Synechocystis,* lanes 7-9 hold colony PCR samples from the same 3 independent colonies after replica plating onto a BG11 chloramphenicol and spectinomycin plate. D) An agarose gel confirming the insertion of the chloramphenicol resistance marker into the *Synechocystis* genome. Isolated gDNA was used as template for amplification with the leuC_genome_F & leuC_genome_R primer pair expected size for WT is 5328 bp and Δ*leuC* is 4581 bp. E) An agarose gel showing colony PCRs on whole cell boil prep samples, lane 2 holds a negative control of untransformed *Synechocystis* with both primer pairs*,* lanes 3-6 use the same colonies from lanes 6-7 in (D), lanes 3-4 used the primer pair leuC_screen_F & cmR_internal_R expected size 1130 bp, lanes 5-6 used the primer pair leuC_screen_R & cmR_internal_F expected size 1230 bp.


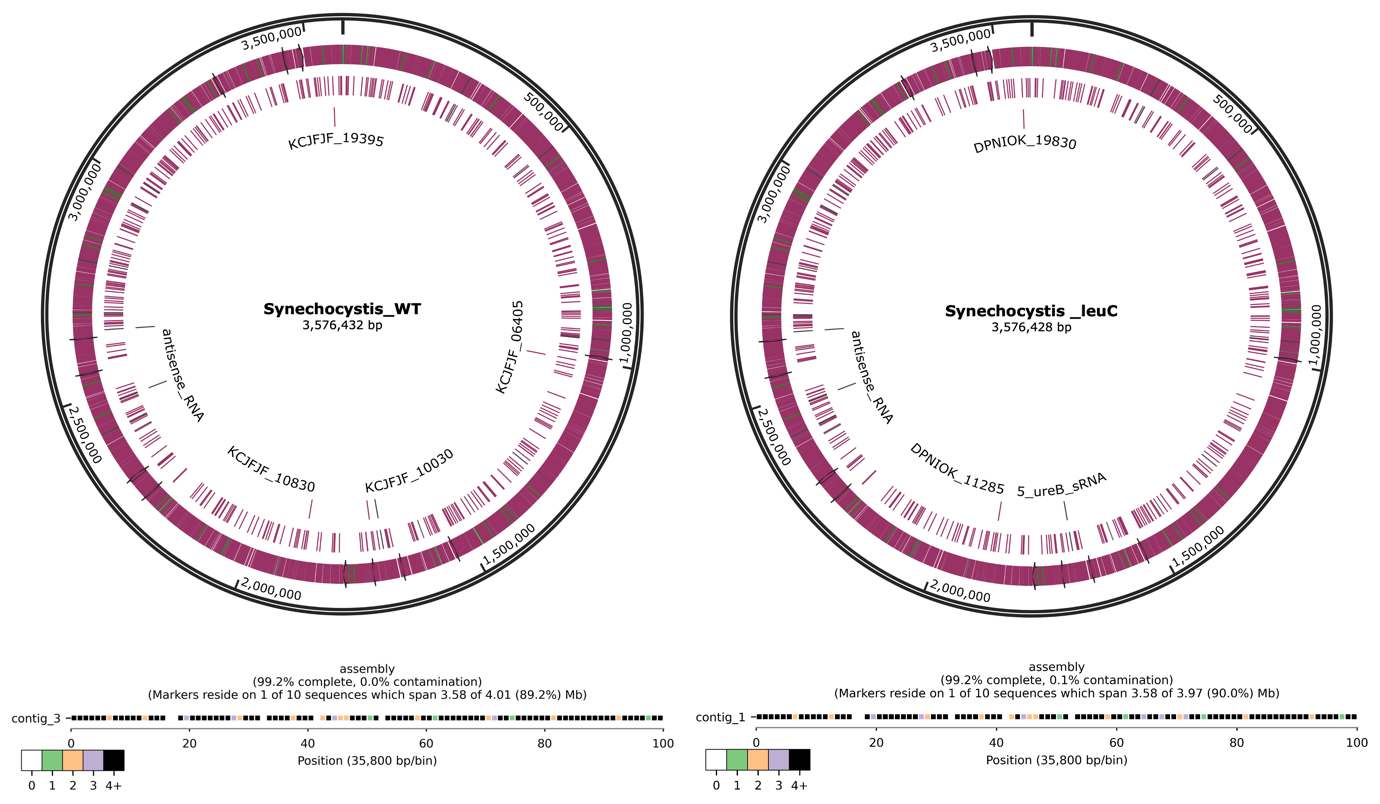


**Supplementary Figure 3:** Consensus genome sequences from Oxford Nanopore data (Plasmidsaurus) of *Synechocystis_WT* (350,952 reads, 309x coverage) prior to any of the changes made and *Synechocystis_*Δ*leuC_pAM2991_cimA (*303,333 reads 257x coverage*)* as used for production of citramalate from this study mapped to NCBI GCF_000009725.1 as a reference genome, created in SnapGene (SnapGene software, www.snapgene.com). Both consensus genomes contain *leuC* 100% identity match to the reference when aligned using Blastn indicating the predominant population is WT. Neither genome contains any significant homology to pAM2991*cimA* when attempting to align with Blastn, indicating the *cimA* expression plasmid has not integrated into the genome of the production strain. There is a slight decrease in coverage and slight indication of contamination in the *Synechocystis_*Δ*leuC_pAM2991_cimA* perhaps indicating the mixed population between Δ*leuC* and WT facilitating the knockdown of *leuC*. The raw sequence read data is available in fastq format as additional files 2 & 3 on OSF https://osf.io/ay52x/ and on Figshare 10.6084/m9.figshare.27170022.

**
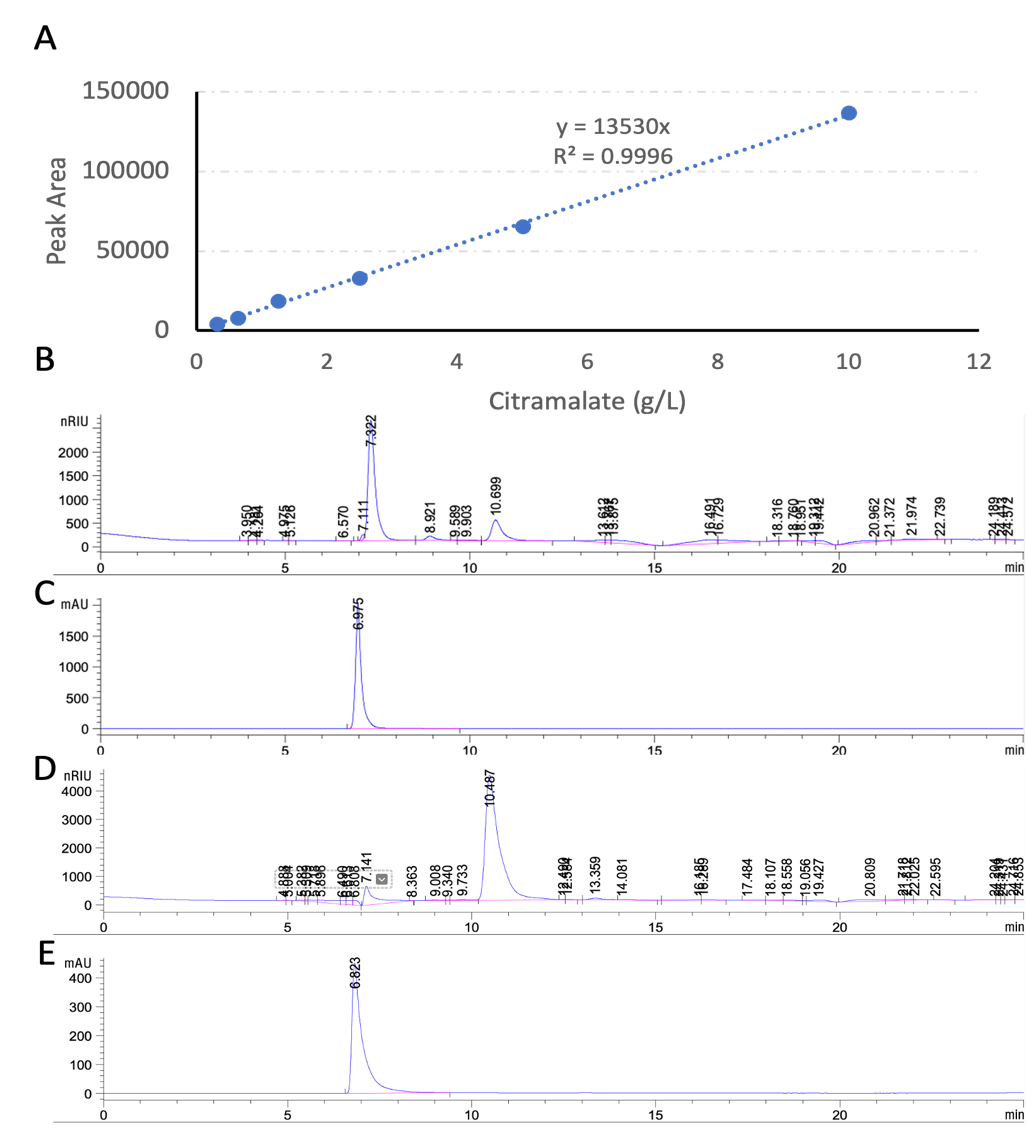
**

**Supplementary Figure 4:** Typical UV-vis Diode Array Detector (DAD) and Refractive Index Detector (RID) plots and a standard curve for the quantification of citramalate by HPLC. A) An example standard curve 0.1-10 g/L from one citramalate quantification by HPLC, a similar standard curve was generated alongside each batch of samples quantified. B) A typical RID trace from an experimental sample of *Synechocystis* in BG11, the peak at 10.699 minutes is citramalate, the peak at 7.322 is also present in BG11 only control samples. C) A typical DAD trace from an experimental sample of *Synechocystis* in BG11. Citramalate has no UV absorption so gives signal, there are no peaks other than the one at 6.975 minutes which is also present in BG11 only control samples. D) A typical RID trace from a standard curve sample of 10 g/L in BG11, the peak at 10.487 minutes is citramalate. E) A typical DAD trace from a standard curve sample of 10 g/L in BG11. Citramalate has no UV absorption so gives signal, there are no peaks other than the one at 6.823 minutes which is also present in BG11 only control samples.


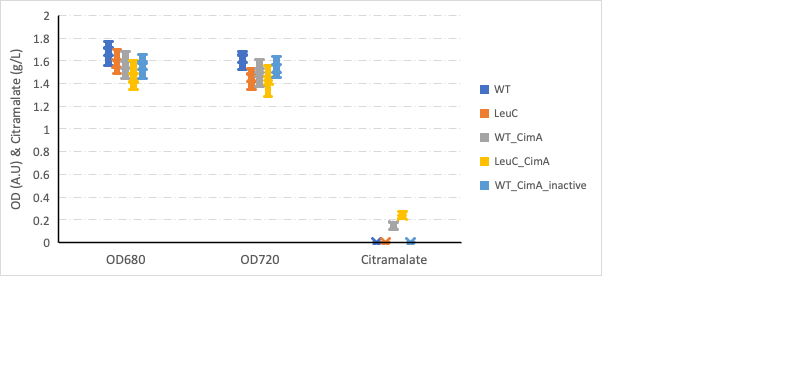


**Supplementary Figure 5**: A box plot of the OD 680 nm & 720 nm, growth, and citramalate titre of the strains tested in this study. Endpoint OD and HPLC measurements after 96 hours 10 ml culture in 25 ml Nunc EasY flasks at 34 °C agitating at 140 RPM with 40 µmol photons m^-2^ s^-1^ warm white light , in BG11 medium. 5 independent colonies of wild type (WT) and Δ*leuC*, with and without pAM2991*CimA*, were assayed for citramalate production under standard photosynthetic growth conditions. We observed citramalate production by all of sub strains with pAM2991*CimA* (*CimA* = 0.152 ± 0.037, Δ*leuC*_pAM2991*CimA* = 0.238 ± 0.032), *Synechocystis*_Δ*leuC*_pAM2991*CimA* accumulated the most citramalate, significantly more than *Synechocystis*_WT_ pAM2991*CimA* by two-tailed T-test assuming equal variance (p = 0.037). We did not observe citramalate accumulation above the detection limit of our HPLC method in controls, strains with no *CimA* or strains with an inactive *CimA* variant ^1^ insert in pAM2991. Without any functional LeuC*, Synechocystis* should be a leucine auxotroph. We did not observe leucine auxotrophy in Δ*leuC* genotypes, nor did we observe fully segregated knock outs, our strain appears to be heterogenous for WT and knock out despite repeated subculturing on selective media. *Synechocystis_* Δ*leuC* was not segregated with leucine supplementation thus full segregation would be fatal; *Synechocystis* is polyploid^2^ thus the Δ*leuC* was knocked-down not knocked-out. This Δ*leuC* knock-down improved citramalate accumulation without impacting upon growth. Optimisation of the ratio of WT to Δ*leuC* and thus the strength of the knock down was outside the scope of this study.


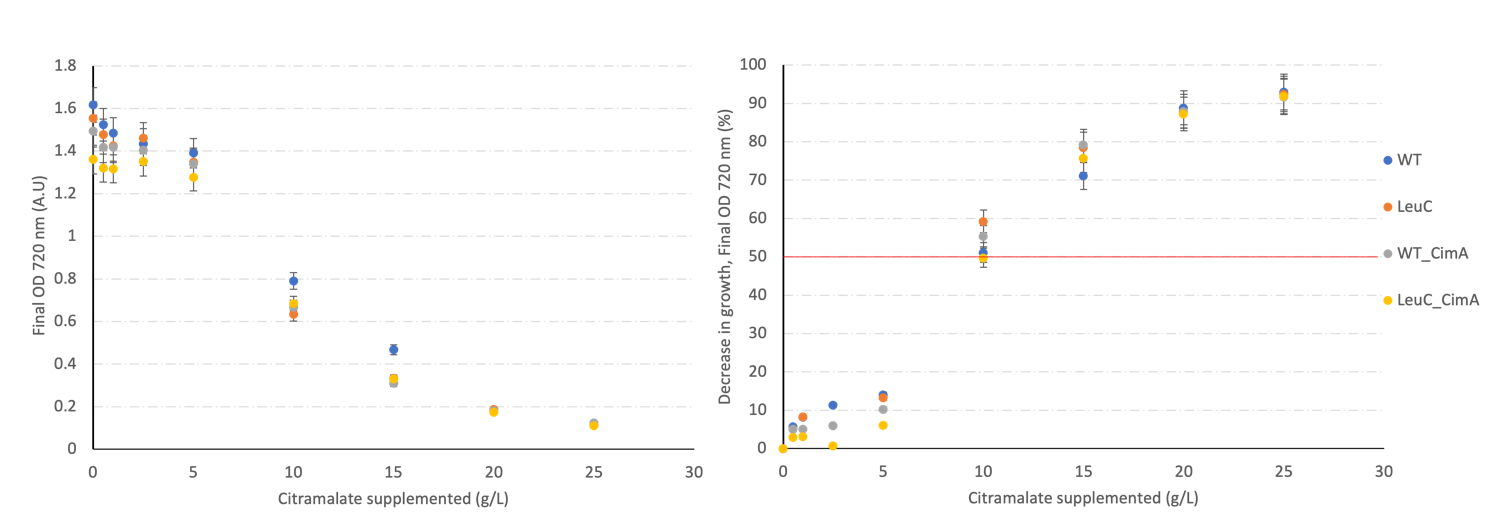


**Supplementary Figure 6**: Endpoint growth measurements (n = 3) after 96 hours with citramalate supplemented (0 to 25 g/L), left final OD 720 nm (A.U.) and right response (%) to citramalate supplementation EC50 or 50% decrease in growth is indicated by the red line. The tolerance of each of the sub strains to exogenous citramalate was determined by measuring growth over 96 hours in BG11 supplemented with citramalate 0 – 25 g/L. EC50 was ~11.93 g/L. There was no discernible difference in the growth of any sub-strain with citramalate supplements 0-5 g/L. Supplementation of citramalate >10 g/L began to markedly reduce growth of all sub strains, and no growth was observed in any sub strain with >20 g/L, with no significant difference between OD 720 nm at inoculation and after 96 hours for any strain (0.15 ± 0.016 and 0.12 ± 0.014 ) by two tailed T-test assuming equal variance (p = 0.245).


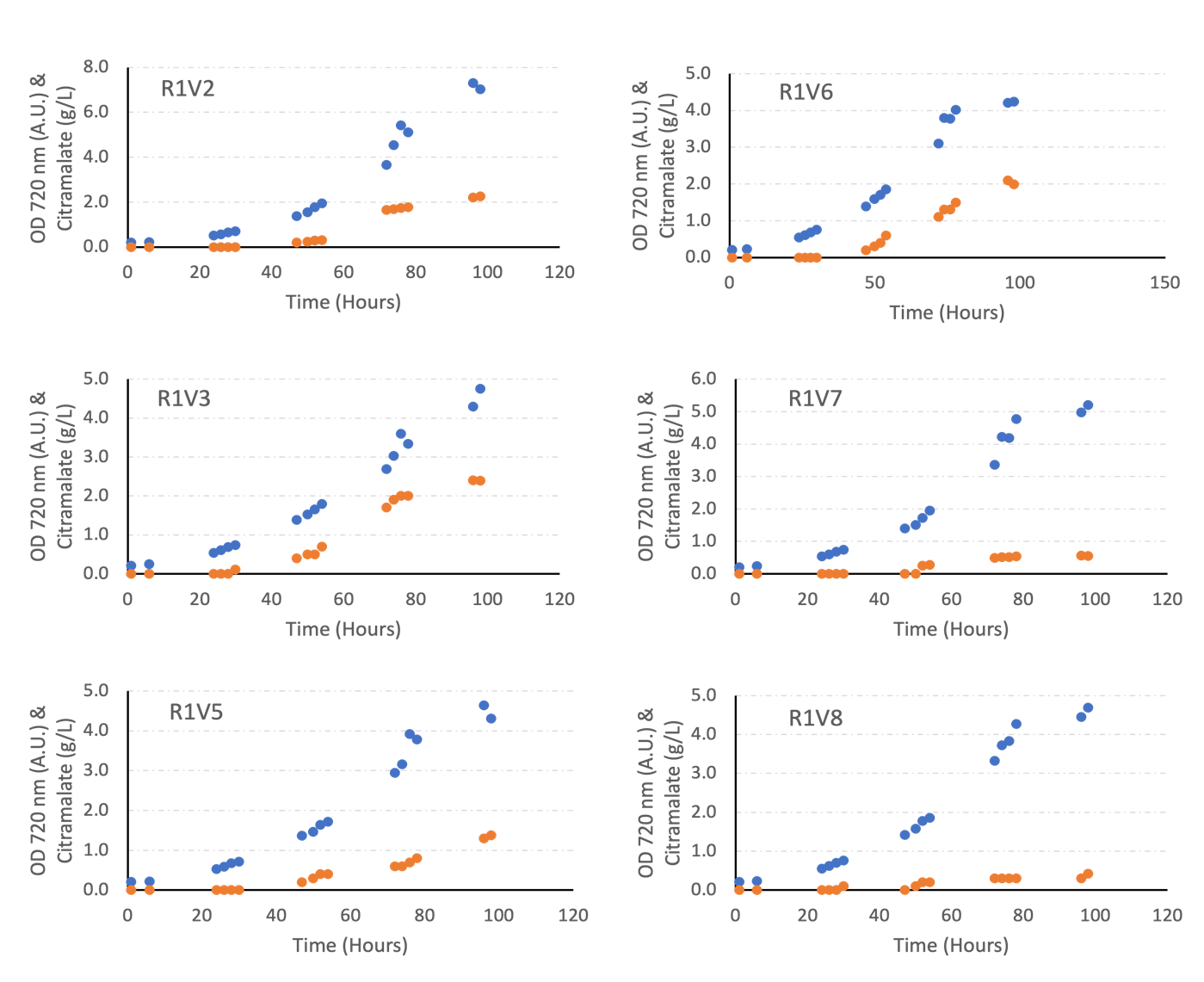


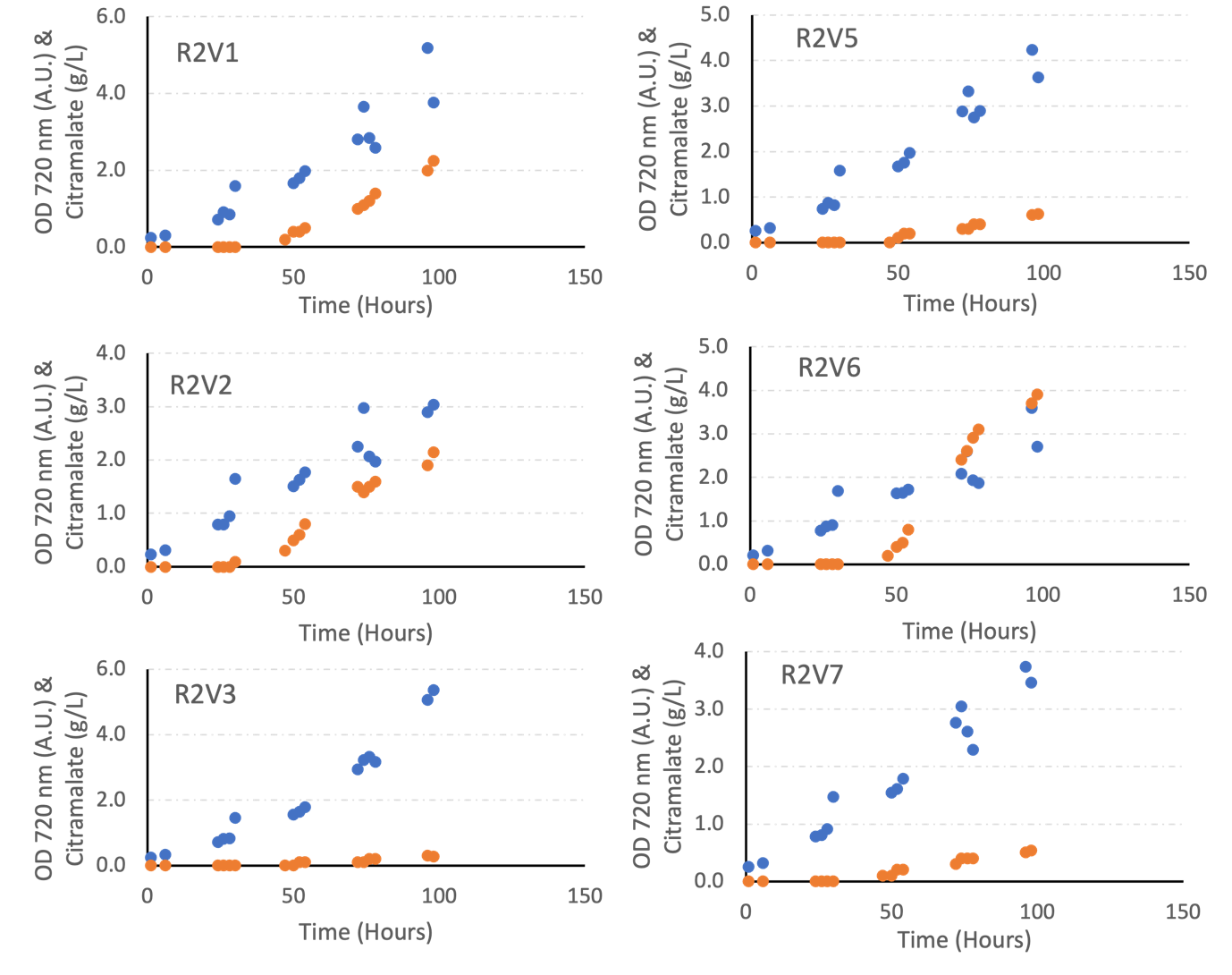


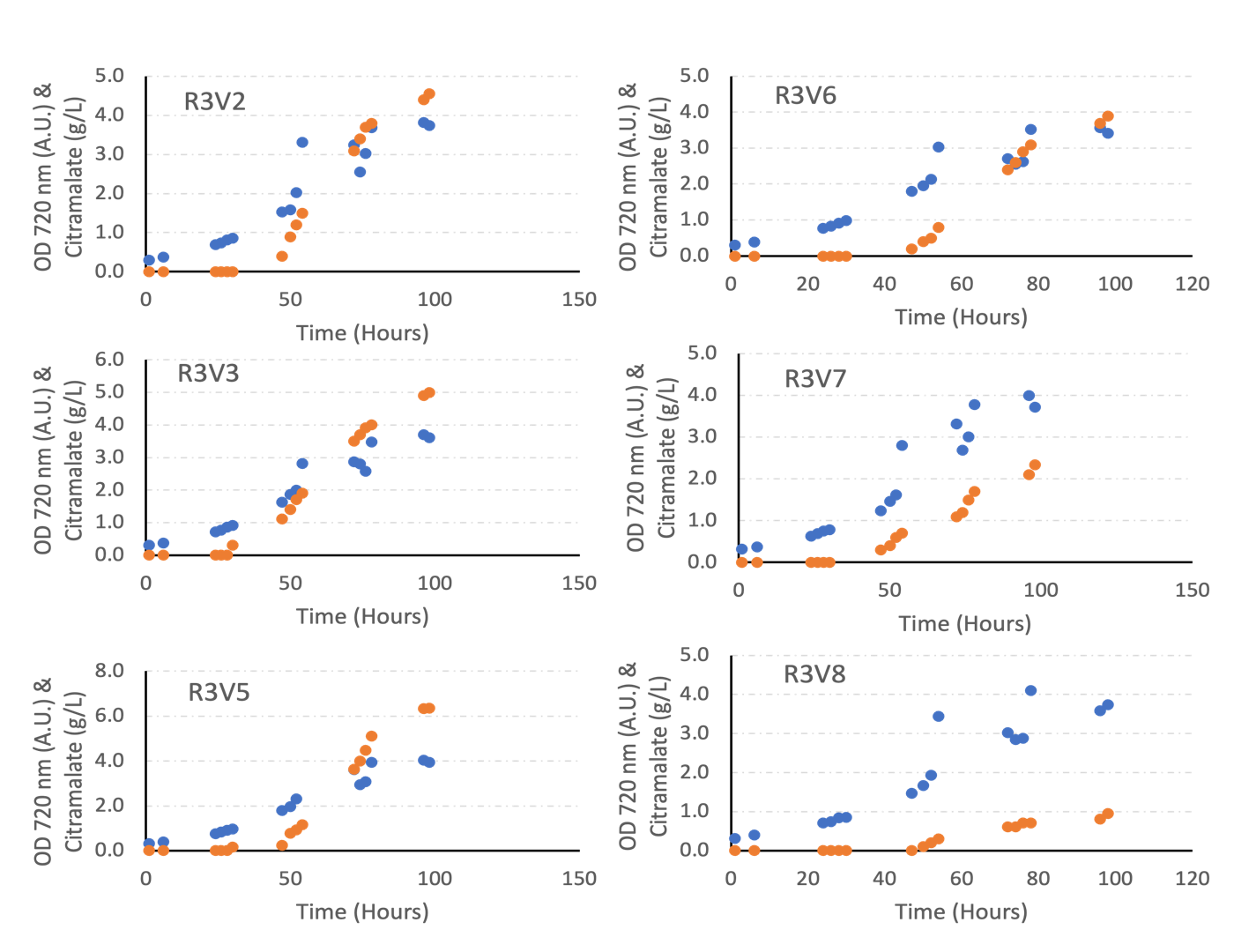


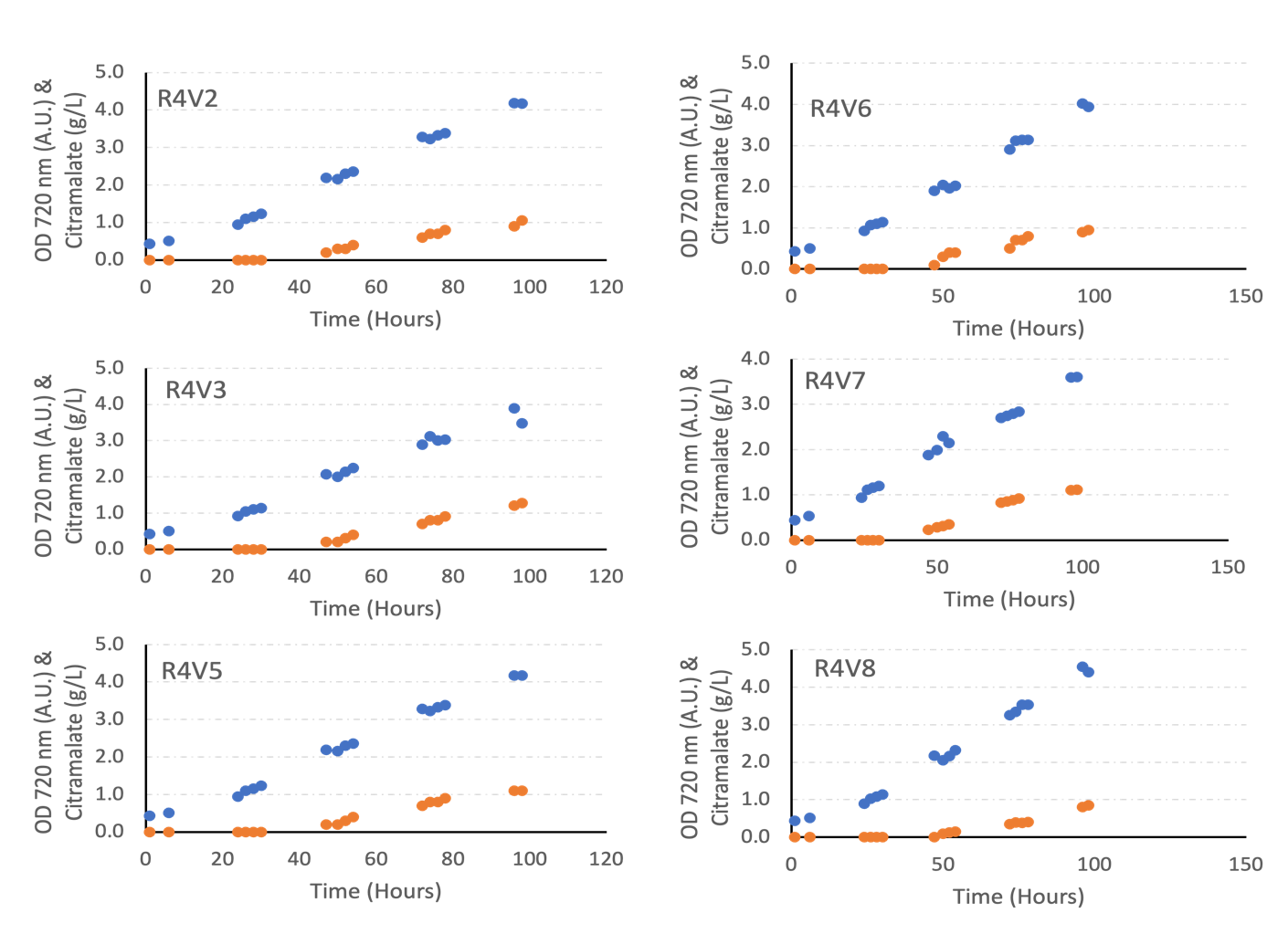


**Supplementary Figure 7**: The growth in blue OD 720 nm (A.U), and citramalate production in orange (g/L), curves for all 24 vessels in the DOE. Each plot is labelled top left with the run and vessel ID, for example run 1 vessel 1 is R1V1. The exact condition for each vessel can be found by cross referencing with Table 1. In the literature where productivity was assessed over time it appears to start high and decrease over time. For example, Namakoshi et al^3^ reported 2010 mg L^-1^ day^-1^ was reported for the first 24 hours that decreased over time, over 72 hours the rate was 986.6 mg L^-1^ day^-1^. We have observed a similar trend with an initial maximal rate soon after changing conditions evident in most vessels of the DOE, that also tends to decrease over time. Highly productive cultures were also beginning to lose cell viability. Aliquots taken at 96 hours from R3V2, R3V3, R3V5, and R3V6 showed little to no growth when spotted onto BG11 agar plates. Similar tests from all other vessels gave clear growth without signs of contamination.


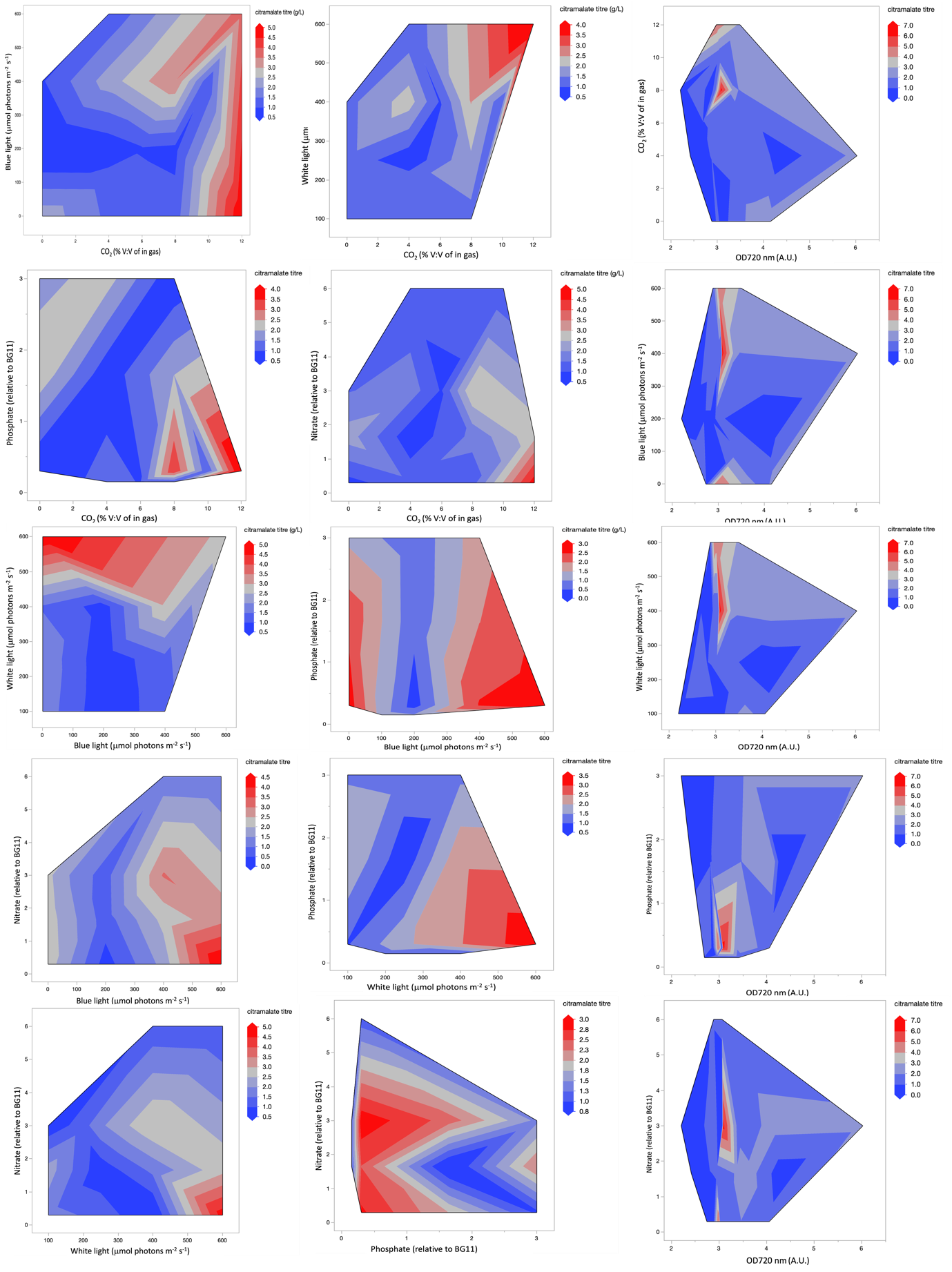


**Supplementary Figure 8**: Contour plots showing citramalate titre for each possible iteration of 2-factor interaction between CO_2_, blue light, white light, phosphate, nitrate, and OD 720 nm. There are clear minima and maxima and clear interactions between some factors. There is a particularly strong correlation between growth and citramalate titre as indicated by the tight maxima around OD 720 nm ~3 in all contour plots. There are clear optima maxima for example when blue and/or white light is high around high CO_2_ ~ 8 %, low phosphate ~0.2 or 1/5^th^ the concentration of BG 11 medium (0.076 mM K_2_HPO_4_), low nitrate ~0.5 - 1 or 8.8 - 17.6 mM. Some plots have no clear maxima show no correlation between the two variables and titre, such as the plot of phosphate, nitrate, and citramalate titre. From these contour plots we can conclude that there are complex multi-factor interactions determining citramalate titre and no single variable tested is determinant for the observed titres.


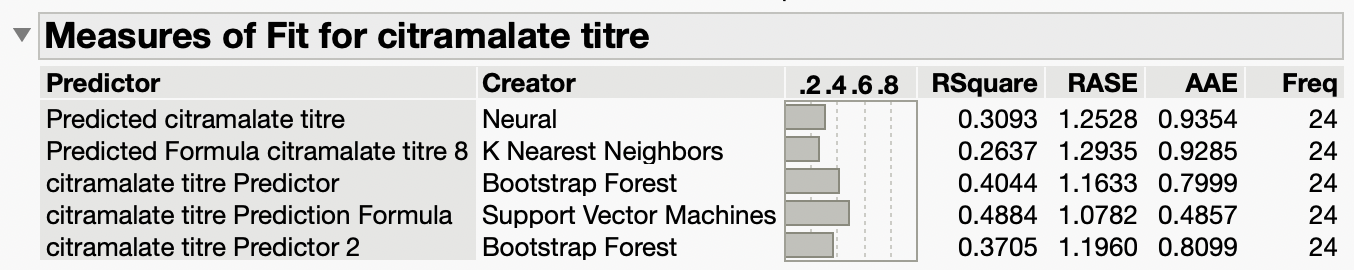

**Supplementary Figure 9**: A summary of the models tested in JMP and the weighting of the DOE factors in the equation of the support vector machines model given as ‘T#6’. We made a predictive model to better examine the complex nature of the multi-factor interactions determining citramalate titre, and to extrapolate from the conditions tested experimentally. The experimental data was fitted to all the models available in JMP (JMP Pro 16) using default parameters, excluding any that fit only when excluding 1 or more of our 24 data points as outliers. The support vector machines had the highest R^2^, 0.4884, and the best fit for a predicted vs actual citramalate titre plot (Main text Figure 4).

**Additional file: 1 Tables**

**Supplementary table 1**: Primers used in this study

| **Primer** | **Purpose** | **Sequence** |
| --- | --- | --- |
| cimA_F | To amplify *cimA* from pET20b with overhang for InFusion into linearised pAM2991 | ggattcacacaggaaacagaccgaattctaatgatgatttgtatcctcta |
| cimA_R | To amplify *cimA* from pET20b with overhang for InFusion into linearised pAM2991 | gtttacaagcatactagaggatcggcggccgctattacaattcacctcta |
| cimA_screen_F | To amplify the MCS site of pAM2991 and verify *cimA* insertion | ggataatgttttttgcgccgac |
| cimA_screen_R | To amplify the MCS site of pAM2991 and verify *cimA* insertion | ctaagctgatccggtgg |
| Lac_operator_F | To amplify near the MCS site and pTRC of pAM2991 and remove the Lac operator by InFusion | cacacattatacgagccggatgattaattgtcaa |
| Lac_operator_R | To amplify near the MCS site and pTRC of pAM2991 and remove the Lac operator by InFusion | tccggctcgtataatgtgtgcacacaggaaacagaccatggaatt |
| Vec_Pam2991_leuC_Chl_F | To amplify and linearise pAM2991 for InFusion insertion of Δ*leuC* 3 fragments | gatgaaaacgaaacggatgaaggcacgaacc |
| Vec_Pam2991_leuC_Chl_R | To amplify and linearise pAM2991 for InFusion insertion of Δ*leuC* 3 fragments | aagtggcaccgtcgcggcgctt |
| H1_F | To amplify homology arm 1 from the *Synechocystis* genome with overhang for InFusion into pAM2991 | gcgacggtgccacttgtatcgggtccag |
| H1_R | To amplify homology arm 1 from the *Synechocystis* genome with overhang for InFusion into pAM2991 | agcggggccgccgagggaaaatgaatggaga |
| H2_R | To amplify homology arm 2 from the *Synechocystis* genome with overhang for InFusion into pAM2991 | catccgtttcgttttcatcgagagtggtggtggaatc |
| H2_F | To amplify homology arm 2 from the *Synechocystis* genome with overhang for InFusion into pAM2991 | tcgccagcatcttcatccgaccaaacaatcac |
| cmR_F | To amplify *cmR* from pSEVA321 with overhang for InFusion into pAM2991 | ctcggcggccccgctgactagtgc |
| cmR_R | To amplify *cmR* from pSEVA321 with overhang for InFusion into pAM2991 | tgaagatgctggcgaaaatgagacgttgat |
| leuC_screen_F | To amplify the 3-fragment insertion site of pAM2991 and verify H1_*cmR*_H2 insertion and presence of pAM2991_ Δ*leuC* | ccagaaaaccgaggatgcg |
| leuC_screen_R | To amplify the 3-fragment insertion site of pAM2991 and verify H1_*cmR*_H2 insertion insertion and presence of pAM2991_ Δ*leuC* | gcgagggctttactaagctg |
| cmR_internal_F | To amplify from inside the *cmR* gene and confirm location with leuC_genome_R or cimA_screen_R | gccattgggatatatcaacggtgg |
| cmR_internal_R | To amplify from inside the *cmR* gene and confirm location with leuC_genome_F or cimA_screen_F | tgtgatggcttccatgtcggc |
| leuC_genome_F | To amplify from outside homology arm 1 on the *Synechocystis* genome with leuC_internal_R to confirm the presence of *leuC* or *cmR* | ttagtgattttttcgggcacc |
| leuC_genome_R | To amplify from outside homology arm 1 on the *Synechocystis* genome with leuC_internal_F to confirm the presence of *leuC* or *cmR* | cggaaagttagctgatgcgact |

**Supplementary table 2:** Plasmid sequences, these sequences were confirmed on plasmids isolated from *Synechocystis* using the “standard high concentration plasmid” sequencing service performed by Plasmidsaurus using Oxford Nanopore Technology with custom analysis and annotation.

| **Plasmid** | **Sequence (backbone-lowercase, insert-uppercase)** |
| --- | --- |
| pAM2991*cimA* | actcaccagtcacagaaaagcatcttacggatggcatgacagtaagagaattatgcagtgctgccataaccatgagtgataacactgcggccaacttacttctgacaacgatcggaggaccgaaggagctaaccgcttttttgcacaacatgggggatcatgtaactcgccttgatcgttgggaaccggagctgaatgaagccataccaaacgacgagcgtgacaccacgatgcctgcagcaatggcaacaacgttgcgcaaactattaactggcgaactacttactctagcttcccggcaacaattaatagactggatggaggcggataaagttgcaggaccacttctgcgctcggcccttccggctggctggtttattgctgataaatctggagccggtgagcgtgggtctcgcggtatcattgcagcactggggccagatggtaagccctcccgtatcgtagttatctacacgacggggagtcaggcaactatggatgaacgaaatagacagatcgctgagataggtgcctcactgattaagcattggtaactgtcagaccaagtttactcatatatactttagattgatttaaaacttcatttttaatttaaaaggatctaggtgaagatcctttttgataatctcatgaccaaaatcccttaacgtgagttttcgttccactgagcgtcagaccccgtagaaaagatcaaaggatcttcttgagatcctttttttctgcgcgtaatctgctgcttgcaaacaaaaaaaccaccgctaccagcggtggtttgtttgccggatcaagagctaccaactctttttccgaaggtaactggcttcagcagagcgcagataccaaatactgtccttctagtgtagccgtagttaggccaccacttcaagaactctgtagcaccgcctacatacctcgctctgctaatcctgttaccagtggctgctgccagtggcgataagtcgtgtcttaccgggttggactcaagacgatagttaccggataaggcgcagcggtcgggctgaacggggggttcgtgcacacagcccagcttggagcgaacgacctacaccgaactgagatacctacagcgtgagctatgagaaagcgccacgcttcccgaagggagaaaggcggacaggtatccggtaagcggcagggtcggaacaggagagcgcacgagggagcttccagggggaaacgcctggtatctttatagtcctgtcgggtttcgccacctctgacttgagcgtcgatttttgtgatgctcgtcaggggggcggagcctatggaaaaacgccagcaacgcggcctttttacggttcctggccttttgctggccttttgctcacatgttctttcctgcgttatcccctgattctgtggataaccgtattaccgcctttgagtgagctgataccgctcgccgcagccgaacgaccgagcgcagcgagtcagtgagcgaggaagcggaagagcgcctgatgcggtattttctccttacgcatctgtgcggtatttcacaccgcatatggtgcactctcagtacaatctgctctgatgccgcatagttaagccagtatacactccgctatcgctacgtgactgggtcatggctgcgccccgacacccgccaacacccgctgacgcgccctgacgggcttgtctgctcccggcatccgcttacagacaagctgtgaccgtctccgggagctgcatgtgtcagaggttttcaccgtcatcaccgaaacgcgcgaggcaggatcgatccggcagccggcggagcgctgctttcttggcaagcggtcgccagccccaacgccagggctgccagcccgaaacagcggggcaaggcagcttggaagggcgatcgcagcacgggcatggcaatgtctctctgaaggaatgcagaccttattcgtacagccagggttgaatcgtgggggtccaatcacttagctctgctgggctaaaccagagagcaatttcctgttgtgctgtttcgattgcatccgagccatggatgatgttgcggccaatattgacaccaaaatcaccacggatggtgcccggttctgccgtcagcggattggtagcgccgatcaacttgcgagcagccgccacaacgccttcgccttccaagacgatcgccacgatcggcccagaggtgatgaactcgacgaggccattgaagaaggggcgctcgcggtggacagcatagtgctgttcggccagctcgcgactgggcttcagctgctttaggcccaccagtttgaagcctttttgctcaaagcggccgatgatcgtaccgaccaaaccccgctgaacgccatcgggcttgatggcaataaatgtgcgttccacagacatctagatagtcctcaagacgaggcaagcattgagcttgccttcctatggttcgggatcactgggattcttgacaagcgatcgcggtcacatcgctatctcttaggacttcgcagcgggcgagtcggattgacccggtagggatttcgccagatcaatgcccgtggtttgtttcagcttctccagcaagctagcgatttgggtagcgctgccttccccttcgccaatcacagtgatcgactccacgtcgatatctggcacggtgcctgaaagcgtgacgagcagggactcgaagcttgcatgcctgcaggtcgactctagagctttatgcttgtaaaccgttttgtgaaaaaatttttaaaataaaaaaggggacctctagggtccccaattaattagtaatataatctattaaaggtcattcaaaaggtcatccaccggatcaattcccctgctcgcgcaggctgggtgccaagctctcgggtaacatcaaggcccgatccttggagcccttgccctcccgcacgatgatcgtgccgtgatcgaaatccagatccttgacccgcagttgcaaaccctcactgatccgcatgcccgttccatacagaagctgggcgaacaaacgatgctcgccttccagaaaaccgaggatgcgaaccacttcatccggggtcagcaccaccggcaagcgccgcgacggccgaggtcttccgatctcctgaagccagggcagatccgtgcacagcaccttgccgtagaagaacagcaaggccgccaatgcctgacgatgcgtggagaccgaaaccttgcgctcgttcgccagccaggacagaaatgcctcgacttcgctgctgcccaaggttgccgggtgacgcacaccgtggaaacggatgaaggcacgaacccagtggacataagcctgttcggttcgtaagctgtaatgcaagtagcgtatgcgctcacgcaactggtccagaaccttgaccgaacgcagcggtggtaacggcgcagtggcggttttcatggcttgttatgactgtttttttggggtacagtctatgcctcgggcatccaagcagcaagcgcgttacgccgtgggtcgatgtttgatgttatggagcagcaacgatgttacgcagcagggcagtcgccctaaaacaaagttaaacatcatgagggaagcggtgatcgccgaagtatcgactcaactatcagaggtagttggcgtcatcgagcgccatctcgaaccgacgttgctggccgtacatttgtacggctccgcagtggatggcggcctgaagccacacagtgatattgatttgctggttacggtgaccgtaaggcttgatgaaacaacgcggcgagctttgatcaacgaccttttggaaacttcggcttcccctggagagagcgagattctccgcgctgtagaagtcaccattgttgtgcacgacgacatcattccgtggcgttatccagctaagcgcgaactgcaatttggagaatggcagcgcaatgacattcttgcaggtatcttcgagccagccacgatcgacattgatctggctatcttgctgacaaaagcaagagaacatagcgttgccttggtaggtccagcggcggaggaactctttgatccggttcctgaacaggatctatttgaggcgctaaatgaaaccttaacgctatggaactcgccgcccgactgggctggcgatgagcgaaatgtagtgcttacgttgtcccgcatttggtacagcgcagtaaccggcaaaatcgcgccgaaggatgtcgctgccgactgggcaatggagcgcctgccggcccagtatcagcccgtcatacttgaagctagacaggcttatcttggacaagaagaagatcgcttggcctcgcgcgcagatcagttggaagaatttgtccactacgtgaaaggcgagatcaccaaggtagtcggcaaataatgtctaacaattcgttcaagccgacgccgcttcgcggcgcggcttaactcaagcgttagatgcactaagcacataattgctcacagccaaactatcaggtcaagtctgcttttattatttttaagcgtgcataataagccctacacaaattgggagatatatcatgaaaggctggctttttcttgttatcgcaatagttggcgaagtaatcgcaacatccgcattaaaatctagcgagggctttactaagctgatccggtggatgaccttttgaatgacctttaatagattatattactaattaattggggaccctagaggtccccttttttattttaaaaattttttcacaaaacggtttacaagcatactagaggatcggcggccgctaTTACAATTCACCTCTAACCTCACTCACGACTGCCAGCAGATCCGGGTGCGGGACGTTTTCACGCAAGTCGCTGAATTTTTTCACACCTTTGTAGATTTTGTGCAACTGCTCGTCGGCAATGTAGATGCACATCAGGTGCAGTTTGTATTACAGCGCTTCACGGCAGCCGTCTTTACACAAAACGACACCACTACAGTGACTCACCACTTCCGCCTGGATCGCTTGGTGGGGCTGGGAGTGTTCAACCAAGCAGTAAATGTCGACACCCGGCTAATGCGTAAGTGTGTAGTTACTAATAATCGTTTTGTACGGCGACATCGGCAACTGCACCAGACCGCTAACGACACAGCACATCTCGTTCAGCTCTTCCACCTCGATCTAGGGGTAGTTGCAGTTCAAGACTTTGGGCGGCGAAATCACTTTTTTCAACGCCGCGTAGCCTGTACCTTTACAAATGCGGTACACGGCAATGTGGCTCTCAATCGCGCGACCCAGCATCGACGAGCCCGAGTATGTGGCCGGCACGCTGAAATTGTGATGGCCGTACATGCTAATCGACAGGTTAACGTTTTTGGAGATTTTCTGGAGCAGTTACTCCGGCTATTGCGAGGCCAGAACACAAACGGAGTCACGAATGCCAATACGGTTCGAACCAACCTCCTGGCGTTAGTAGAGCAATTCGACCAAGAAGTCCATGTCGCAGCCGGACGAGTTTTCAGTCGGCAACTGAATGACCAAACGCTACTGTTTTGTGTCCTGCACCGATTTCAGTCCGGGAATCAACATTTTGTATTCGGGCTGGCTCAACTAGTTTTCCATGTTGACCGGCGGGGCCGGAATAATCAGGTGCATCGGGTGCACATCGCCCTCCAGCGACGTATTAATGTGCATCGTCAACGGACGAATGAGCGTACAAATTTACGGGTGCAGGCCTTCTTGGGCAACCAATTCAACGCACTCGCCCTGGCTTTCCGAGGTAACTGCCGGACGTGCTTTGACAATGTCCATGCTCAATTTATCCAGTTTCTCCGCGACTTGCAGCTCATTGTACGGAGTCAGGCGAACACCCGGGGACTATTAGCTGTCGCCCAAGGATCTGTAGAGGATACAAATCATCATtagaattcggtctgtttcctgtgtgaatccacacattatacgagccggatgattaattgtcaacagctcatttcagaatatttgccagaaccgttatgatgtcggcgcaaaaaacattatccagaacgggagtgcgccttgagcgacacgaattatgcagtgatttacgacctgcacagccataccacagcttccgatggctgcctgacgccagaagcattggtgcaccgtgcagtcgatgataagctgtcaaaccagatcaattcgcgctaactcacattaattgcgttgcgctcactgcccgctttccagtcgggaaacctgtcgtgccagctgcattaatgaatcggccaacgcgcggggagaggcggtttgcgtattgggcgccagggtggtttttcttttcaccagtgagacgggcaacagctgattgcccttcaccgcctggccctgagagagttgcagcaagcggtccacgctggtttgccccagcaggcgaaaatcctgtttgatggtggttgacggcgggatataacatgagctgtcttcggtatcgtcgtatcccactaccgagatatccgcaccaacgcgcagcccggactcggtaatggcgcgcattgcgcccagcgccatctgatcgttggcaaccagcatcgcagtgggaacgatgccctcattcagcatttgcatggtttgttgaaaaccggacatggcactccagtcgccttcccgttccgctatcggctgaatttgattgcgagtgagatatttatgccagccagccagacgcagacgcgccgagacagaacttaatgggcccgctaacagcgcgatttgctggtgacccaatgcgaccagatgctccacgcccagtcgcgtaccgtcttcatgggagaaaataatactgttgatgggtgtctggtcagagacatcaagaaataacgccggaacattagtgcaggcagcttccacagcaatggcatcctggtcatccagcggatagttaatgatcagcccactgacgcgttgcgcgagaagattgtgcaccgccgctttacaggcttcgacgccgcttcgttctaccatcgacaccaccacgctggcacccagttgatcggcgcgagatttaatcgccgcgacaatttgcgacggcgcgtgcagggccagactggaggtggcaacgccaatcagcaacgactgtttgcccgccagttgttgtgccacgcggttgggaatgtaattcagctccgccatcgccgcttccactttttcccgcgttttcgcagaaacgtggctggcctggttcaccacgcgggaaacggtctgataagagacaccggcatactctgcgacatcgtataacgttactggtttcacattcaccaccctgaattgactctcttccgggcgctatcatgccataccgcgaaaggttttgcaccattcgatggtgtcaacgtaaatgcatgccgcttcgccttcgcgcgcgaattgatctgctgcctcgcgcgtttcggtgatgacggtgaaaacctctgacacatgcagctcccggagacggtcacagcttgtctgtaagcggatgccgggagcagacaagcccgtcagggcgcgtcagcgggtgttggcgggtgtcggggcgcagccatgacccgcggcccagatccccgggtaccgagctcgaatttcgagcttctggagcaggaagatgtcgcgggcattagcaccagcggtctgccaagcctccgccagccgttgggtcccttccgcttgagcttttccatcttcgacgatacgggcggcggccccccgcgcttccgcgatcgcccgtttacaagctgcctcagctggggcgatcacatcggcttgaagttgctgctgcacctgtttgatccgctcctgctgcacagggagttctgcttggctacgagcgacttcggtagcaatgtccgcttcagcttcggccaccaccgcttcgcgccgcgtcaacgcatcctgaatccggcgctcggcctcggcttgggcgatcgctacatcgcgatcgatccgacgcagggccgtgatcttgtcattttcggccgtttggatcgcagaggcagcctgggcatcggcttcagcaattcgggcatctcgctgcagatcagcccgctgcttgcgtccactagccgagagataaccgacctcatcggaaatgttctggacttgcagcgtatcgaggactagacccagctgctcaaggtcatcctccgcctcttccagcagacttttggcaaaggcaattttgtcctcgttgatctgctccggcgtgaggctggctaaaacaccacgcaagttgccttcgagggtctccttggcaatttgctcgatttccttacggtttttgccaagcagccgctcgatcgcgttgtggatggtcggttcttccccagcaatcttgatattggcaacgccttcaacagtcaggggaatgccgcccttggagaaggcattggaaacgcgcaactcaatgatcatgttggtcagatccatgcggagcgctttttccagcagaggtacccgcaggctgctgccgcccttgaccaagcgatagccaactcggcggccatcactactgcggcgactactgccagcaaagatcaaaatttcactgggttggcagatgtagtagagattgcgcaggactaagctgccagccccggcg |
| pAM2991  Δ*leuC* | actcaccagtcacagaaaagcatcttacggatggcatgacagtaagagaattatgcagtgctgccataaccatgagtgataacactgcggccaacttacttctgacaacgatcggaggaccgaaggagctaaccgcttttttgcacaacatgggggatcatgtaactcgccttgatcgttgggaaccggagctgaatgaagccataccaaacgacgagcgtgacaccacgatgcctgcagcaatggcaacaacgttgcgcaaactattaactggcgaactacttactctagcttcccggcaacaattaatagactggatggaggcggataaagttgcaggaccacttctgcgctcggcccttccggctggctggtttattgctgataaatctggagccggtgagcgtgggtctcgcggtatcattgcagcactggggccagatggtaagccctcccgtatcgtagttatctacacgacggggagtcaggcaactatggatgaacgaaatagacagatcgctgagataggtgcctcactgattaagcattggtaactgtcagaccaagtttactcatatatactttagattgatttaaaacttcatttttaatttaaaaggatctaggtgaagatcctttttgataatctcatgaccaaaatcccttaacgtgagttttcgttccactgagcgtcagaccccgtagaaaagatcaaaggatcttcttgagatcctttttttctgcgcgtaatctgctgcttgcaaacaaaaaaaccaccgctaccagcggtggtttgtttgccggatcaagagctaccaactctttttccgaaggtaactggcttcagcagagcgcagataccaaatactgtccttctagtgtagccgtagttaggccaccacttcaagaactctgtagcaccgcctacatacctcgctctgctaatcctgttaccagtggctgctgccagtggcgataagtcgtgtcttaccgggttggactcaagacgatagttaccggataaggcgcagcggtcgggctgaacggggggttcgtgcacacagcccagcttggagcgaacgacctacaccgaactgagatacctacagcgtgagctatgagaaagcgccacgcttcccgaagggagaaaggcggacaggtatccggtaagcggcagggtcggaacaggagagcgcacgagggagcttccagggggaaacgcctggtatctttatagtcctgtcgggtttcgccacctctgacttgagcgtcgatttttgtgatgctcgtcaggggggcggagcctatggaaaaacgccagcaacgcggcctttttacggttcctggccttttgctggccttttgctcacatgttctttcctgcgttatcccctgattctgtggataaccgtattaccgcctttgagtgagctgataccgctcgccgcagccgaacgaccgagcgcagcgagtcagtgagcgaggaagcggaagagcgcctgatgcggtattttctccttacgcatctgtgcggtatttcacaccgcatatggtgcactctcagtacaatctgctctgatgccgcatagttaagccagtatacactccgctatcgctacgtgactgggtcatggctgcgccccgacacccgccaacacccgctgacgcgccctgacgggcttgtctgctcccggcatccgcttacagacaagctgtgaccgtctccgggagctgcatgtgtcagaggttttcaccgtcatcaccgaaacgcgcgaggcaggatcgatccggcagccggcggagcgctgctttcttggcaagcggtcgccagccccaacgccagggctgccagcccgaaacagcggggcaaggcagcttggaagggcgatcgcagcacgggcatggcaatgtctctctgaaggaatgcagaccttattcgtacagccagggttgaatcgtgggggtccaatcacttagctctgctgggctaaaccagagagcaatttcctgttgtgctgtttcgattgcatccgagccatggatgatgttgcggccaatattgacaccaaaatcaccacggatggtgcccggttctgccgtcagcggattggtagcgccgatcaacttgcgagcagccgccacaacgccttcgccttccaagacgatcgccacgatcggcccagaggtgatgaactcgacgaggccattgaagaaggggcgctcgcggtggacagcatagtgctgttcggccagctcgcgactgggcttcagctgctttaggcccaccagtttgaagcctttttgctcaaagcggccgatgatcgtaccgaccaaaccccgctgaacgccatcgggcttgatggcaataaatgtgcgttccacagacatctagatagtcctcaagacgaggcaagcattgagcttgccttcctatggttcgggatcactgggattcttgacaagcgatcgcggtcacatcgctatctcttaggacttcgcagcgggcgagtcggattgacccggtagggatttcgccagatcaatgcccgtggtttgtttcagcttctccagcaagctagcgatttgggtagcgctgccttccccttcgccaatcacagtgatcgactccacgtcgatatctggcacggtgcctgaaagcgtgacgagcagggactcgaagcttgcatgcctgcaggtcgactctagagctttatgcttgtaaaccgttttgtgaaaaaatttttaaaataaaaaaggggacctctagggtccccaattaattagtaatataatctattaaaggtcattcaaaaggtcatccaccggatcaattcccctgctcgcgcaggctgggtgccaagctctcgggtaacatcaaggcccgatccttggagcccttgccctcccgcacgatgatcgtgccgtgatcgaaatccagatccttgacccgcagttgcaaaccctcactgatccgcatgcccgttccatacagaagctgggcgaacaaacgatgctcgccttccagaaaaccgaggatgcgaaccacttcatccggggtcagcaccaccggcaagcgccgcgacggTGCCACTTGTATCGGGTCCAGCCCTTGGTACTGCCATTGCTGACATTGACGTAAAACTGTTTCAATTACCCATTCCCCCACCACGCTGATAAAGCCAGTTTTTTCGGCGATCGGGATGAAAAGTCCCGGCGAAACAAAACCTTTACGGGGATGGTGCCAACGGATCAAGGCTTCACAGCCGGCCAAACAATGGTTGTGGATAGATACTTTGGGTTGGTAGAAGAGAAAAACTTGCCCAGTGTTTAAACCCACTTCTAAATCCCGCCAAATTTCCGCTTCATCCGGGGTGAGGGTCGGTGTACCGATGGTACCAGAACGGCGTGGCCTCTCGGTCAAGGAACTAGATATACTCCCCTGATAAAAGGCTGACAACTTGGCTTGTTTTTGCAGACGGCTATTAACCGCATTCAGTAAATCTTCTTGTTTAAAAGGCTTAATTAAGTAATCATCCGCGCCGGAATTCATGCCCTTACGAAAATCCTGCATAGTACCCAGAGCTGTCAGAAAAATGAAGGGAATAGCCGCGGTTTTGGCGTTCTTTTGCAGGGCTGTAATCAAGCCATGGCCATCCATTTCCGGCATCATCACATCACAGATGATCAAGTCGGGCATCACCTCCAAGGAGTTGAGCAGCCCCAGGGCCACCGCCCCATTTTCTGCTTCTAGAATCTTATAATTTTCCAGACTCAGGGTTTCGCCAATTAACTCCCGAATAATGGCTTCATCCTCGACAACTAAAATAGTACTCATAGGGATTAATCAAATCTTTGTCGCGGGCCAGAGGGAGTTTTCAGACAAGCTCTGAGTGACATCCTAGCCTAATTTCCCAGACTTTAAGACCAAATTGACTGGATTTTTCTCCATTATTGTTCCTAGGTTCAGGTTGTCAGGTCAGGCTAAGATGGTGATAGTCGCTACGACCACCACCCCATTCTCCATTCATTTTCCCTCGGCGGCCCCGCTGACTAGTGCTTGGATTCTCACCAATAAAAAACGCCCGGCGGCAACCGAGCGTTCTGAACAAATCCAGATGGAGTTCTGAGGTCATTACTGGATCTATCAACAGGAGTCCAAGCGAGCTCGATATCAAATTACGCCCCGCCCTGCCACTCATCGCAGTACTGTTGTAATTCATTAAGCATTCTGCCGACATGGAAGCCATCACAAACGGCATGATGAACCTGAATCGCCAGCGGCATCAGCACCTTGTCGCCTTGCGTATAATATTTGCCCATGGTGAAAACGGGGGCGAAGAAGTTGTCCATATTGGCCACGTTTAAATCAAAACTGGTGAAACTCACCCAGGGATTGGCTGAGACGAAAAACATATTCTCAATAAACCCTTTAGGGAAATAGGCCAGGTTTTCACCGTAACACGCCACATCTTGCGAATATATGTGTAGAAACTGCCGGAAATCGTCGTGGTATTCACTCCAGAGCGATGAAAACGTTTCAGTTTGCTCATGGAAAACGGTGTAACAAGGGTGAACACTATCCCATATCACCAGCTCACCGTCTTTCATTGCCATACGAAATTCCGGATGAGCATTCATCAGGCGGGCAAGAATGTGAATAAAGGCCGGATAAAACTTGTGCTTATTTTTCTTTACGGTCTTTAAAAAGGCCGTAATATCCAGCTGAACGGTCTGGTTATAGGTACATTGAGCAACTGACTGAAATGCCTCAAAATGTTCTTTACGATGCCATTGGGATATATCAACGGTGGTATATCCAGTGATTTTTTTCTCCATTTTAGCTTCCTTAGCTCCTGAAAATCTCGATAACTCAAAAAATACGCCCGGTAGTGATCTTATTTCATTATGGTGAAAGTTGGAACCTCTTACGTGCCGATCAACGTCTCATTTTCGCCAGCATCTTCATCCGACCAAACAATCACCATCGAGGGGGGGCTGCTTTGTCATAGGCTAGCATAGCTGATTAGCATAACTATGAGACATTAGACTATGCAGTGATCTTGAATAAATTTATCTATACTAGTATCAGGAGTTGCCTGCATTCTCGCTCTATTTAATGCACTAAAATCATGCTCTATATCATTCAAGTCTGGAGAATAAGTTGGCAAAAATAACATAATGTGTCCTGCGGACTCCACTATTTCTCTAATCTTATTTTTTCGATGAATGGGGGGGGGGTTATCCATAATCAAGATTGATTTCATTGTTAATGTTGGTATTAAAAAGAATTTAAGCCATCCTTCAAAACCTGTTGTATTTAAGCTTCCGGTGAATAACATCGGCGCTATCAAGTCTTTTTGCATTTTTCTTTTTCTCGCTACAAGATTTTCTTTTTTCCCTCTTTTCCCCTGCTTTTCTCCATATACTCTTTCTCCCTTTTTAGACCATCCATAAAGTGAGGATGCAAATTTCTCAAAACCAGCTTCGTCTATGAATACTAGACTTTCAATACCGTATATTAAAACTAACTGCCTCAAGATTTTTTGATATTCTGCTCGCTTTTGGGCATCCCTCTCTTGATAAAGTAACTGTTTTTTTTCTAGTAATCTTCATTTTTTTGAATTGATAACTGAGTGAAGCAGAAGTCACACCAAATTTTTTAGCTCTTTCTTTTAGAAGCATATCAGGATTATTTTTCACATCCTCTTCTAATTCTTTTCTGTTTATCTTACGCTGACGGTTCTCCACTTTCGTTGCCGCAAGCTCTTCTCTGCCCAACCAGCGATAAACTGTTGCTCTGCCCACCCTAAATGTTTTCGAAGCCTTTGTGATTCCACCACCACTCTCGATGAAAACgaaacggatgaaggcacgaacccagtggacataagcctgttcggttcgtaagctgtaatgcaagtagcgtatgcgctcacgcaactggtccagaaccttgaccgaacgcagcggtggtaacggcgcagtggcggttttcatggcttgttatgactgtttttttggggtacagtctatgcctcgggcatccaagcagcaagcgcgttacgccgtgggtcgatgtttgatgttatggagcagcaacgatgttacgcagcagggcagtcgccctaaaacaaagttaaacatcatgagggaagcggtgatcgccgaagtatcgactcaactatcagaggtagttggcgtcatcgagcgccatctcgaaccgacgttgctggccgtacatttgtacggctccgcagtggatggcggcctgaagccacacagtgatattgatttgctggttacggtgaccgtaaggcttgatgaaacaacgcggcgagctttgatcaacgaccttttggaaacttcggcttcccctggagagagcgagattctccgcgctgtagaagtcaccattgttgtgcacgacgacatcattccgtggcgttatccagctaagcgcgaactgcaatttggagaatggcagcgcaatgacattcttgcaggtatcttcgagccagccacgatcgacattgatctggctatcttgctgacaaaagcaagagaacatagcgttgccttggtaggtccagcggcggaggaactctttgatccggttcctgaacaggatctatttgaggcgctaaatgaaaccttaacgctatggaactcgccgcccgactgggctggcgatgagcgaaatgtagtgcttacgttgtcccgcatttggtacagcgcagtaaccggcaaaatcgcgccgaaggatgtcgctgccgactgggcaatggagcgcctgccggcccagtatcagcccgtcatacttgaagctagacaggcttatcttggacaagaagaagatcgcttggcctcgcgcgcagatcagttggaagaatttgtccactacgtgaaaggcgagatcaccaaggtagtcggcaaataatgtctaacaattcgttcaagccgacgccgcttcgcggcgcggcttaactcaagcgttagatgcactaagcacataattgctcacagccaaactatcaggtcaagtctgcttttattatttttaagcgtgcataataagccctacacaaattgggagatatatcatgaaaggctggctttttcttgttatcgcaatagttggcgaagtaatcgcaacatccgcattaaaatctagcgagggctttactaagctgatccggtggatgaccttttgaatgacctttaatagattatattactaattaattggggaccctagaggtccccttttttattttaaaaattttttcacaaaacggtttacaagcatactagaggatcggcggccgcggatctgggccgccatgagcggatacatatttgaatgtatttagaaaaataaacaaaagagtttgtagaaacgcaaaaaggccatccgtcaggatggccttctgcttaatttgatgcctggcagtttatggcgggcgtcctgcccgccaccctccgggccgttgcttcgcaacgttcaaatccgctcccggcggatttgtcctactcaggagagcgttcaccgacaaacaacagataaaacgaaaggcccagtctttcgactgagcctttcgttttatttgatgcctggcagttccctactctcgcatggggagaccccacactaccatcggcgctacggcgtttcacttctgagttcggcatggggtcaggtgggaccaccgcgctactgccgccaggcaaattctgttttatcagaccgcttctgcgttctgatttaatctgtatcaggctgaaaatcttctctcatccgccaaaacagccaagcttgcatgcctgcaggtcgactctagaggatccccgggtaccgagctcgaattccatggtctgtttcctgtgtgaaattgttatccgctcacaattccacacattatacgagccggatgattaattgtcaacagctcatttcagaatatttgccagaaccgttatgatgtcggcgcaaaaaacattatccagaacgggagtgcgccttgagcgacacgaattatgcagtgatttacgacctgcacagccataccacagcttccgatggctgcctgacgccagaagcattggtgcaccgtgcagtcgatgataagctgtcaaaccagatcaattcgcgctaactcacattaattgcgttgcgctcactgcccgctttccagtcgggaaacctgtcgtgccagctgcattaatgaatcggccaacgcgcggggagaggcggtttgcgtattgggcgccagggtggtttttcttttcaccagtgagacgggcaacagctgattgcccttcaccgcctggccctgagagagttgcagcaagcggtccacgctggtttgccccagcaggcgaaaatcctgtttgatggtggttgacggcgggatataacatgagctgtcttcggtatcgtcgtatcccactaccgagatatccgcaccaacgcgcagcccggactcggtaatggcgcgcattgcgcccagcgccatctgatcgttggcaaccagcatcgcagtgggaacgatgccctcattcagcatttgcatggtttgttgaaaaccggacatggcactccagtcgccttcccgttccgctatcggctgaatttgattgcgagtgagatatttatgccagccagccagacgcagacgcgccgagacagaacttaatgggcccgctaacagcgcgatttgctggtgacccaatgcgaccagatgctccacgcccagtcgcgtaccgtcttcatgggagaaaataatactgttgatgggtgtctggtcagagacatcaagaaataacgccggaacattagtgcaggcagcttccacagcaatggcatcctggtcatccagcggatagttaatgatcagcccactgacgcgttgcgcgagaagattgtgcaccgccgctttacaggcttcgacgccgcttcgttctaccatcgacaccaccacgctggcacccagttgatcggcgcgagatttaatcgccgcgacaatttgcgacggcgcgtgcagggccagactggaggtggcaacgccaatcagcaacgactgtttgcccgccagttgttgtgccacgcggttgggaatgtaattcagctccgccatcgccgcttccactttttcccgcgttttcgcagaaacgtggctggcctggttcaccacgcgggaaacggtctgataagagacaccggcatactctgcgacatcgtataacgttactggtttcacattcaccaccctgaattgactctcttccgggcgctatcatgccataccgcgaaaggttttgcaccattcgatggtgtcaacgtaaatgcatgccgcttcgccttcgcgcgcgaattgatctgctgcctcgcgcgtttcggtgatgacggtgaaaacctctgacacatgcagctcccggagacggtcacagcttgtctgtaagcggatgccgggagcagacaagcccgtcagggcgcgtcagcgggtgttggcgggtgtcggggcgcagccatgacccgcggcccagatccccgggtaccgagctcgaatttcgagcttctggagcaggaagatgtcgcgggcattagcaccagcggtctgccaagcctccgccagccgttgggtcccttccgcttgagcttttccatcttcgacgatacgggcggcggccccccgcgcttccgcgatcgcccgtttacaagctgcctcagctggggcgatcacatcggcttgaagttgctgctgcacctgtttgatccgctcctgctgcacagggagttctgcttggctacgagcgacttcggtagcaatgtccgcttcagcttcggccaccaccgcttcgcgccgcgtcaacgcatcctgaatccggcgctcggcctcggcttgggcgatcgctacatcgcgatcgatccgacgcagggccgtgatcttgtcattttcggccgtttggatcgcagaggcagcctgggcatcggcttcagcaattcgggcatctcgctgcagatcagcccgctgcttgcgtccactagccgagagataaccgacctcatcggaaatgttctggacttgcagcgtatcgaggactagacccagctgctcaaggtcatcctccgcctcttccagcagacttttggcaaaggcaattttgtcctcgttgatctgctccggcgtgaggctggctaaaacaccacgcaagttgccttcgagggtctccttggcaatttgctcgatttccttacggtttttgccaagcagccgctcgatcgcgttgtggatggtcggttcttccccagcaatcttgatattggcaacgccttcaacagtcaggggaatgccgcccttggagaaggcattggaaacgcgcaactcaatgatcatgttggtcagatccatgcggagcgctttttccagcagaggtacccgcaggctgctgccgcccttgaccaagcgatagccaactcggcggccatcactactgcggcgactactgccagcaaagatcaaaatttcactgggttggcagatgtagtagagattgcgcaggactaagctgccagccccggcg |
